# Genotype × environment interactions underlying geographic divergence in carotenoid accumulation and kernel pigmentation of foxtail millet

**DOI:** 10.1186/s12870-025-08008-1

**Published:** 2026-01-02

**Authors:** Xin Zhao, Yueyue Wang, Ziyi Deng, Zhongxiang Li, Meng Yue, Yiru Zhang, Ming Duan, Xiaodong Liu, Bin Zhang, Siyu Hou, Yushen Wang, Huatao Liu, Wei Zhang, Hui Zhi, Hongying Li, Yuanhuai Han

**Affiliations:** 1 College of Agronomy, Shanxi Agricultural University, Taigu, 030801 China; 2 Shanxi Institute of Organic Dryland Farming, Shanxi Agricultural University, Taiyuan, 030031 China; 3 Key Laboratory of Sustainable Dryland Agriculture (Co-Construction by Ministry of Agriculture and Rural Affairs and Shanxi Province), Taiyuan, 030031 China; 4 College of Biological Sciences and Technology, Taiyuan Normal University, Yuci, 030619 China; 5 Experimental Teaching Center, Shanxi Agricultural University, Taigu, 030801 China; 6 Institute of Crop Sciences, Chinese Academy of Agricultural Sciences, Beijing, 100081 China

**Keywords:** Setaria italica, Nutritional quality, Commercial quality, Genotype, Ecological factor, Metabolomics

## Abstract

**Background:**

Foxtail millet (*Setaria italica* L.), a drought-resistant cereal crop, is nutritionally valued for its carotenoid-rich kernels that significantly influence both commercial quality and health benefits. While carotenoid content and kernel pigmentation are known to be affected by genotype and environment, the mechanisms governing these relationships remain unclear. This study systematically examined the ecological and genetic determinants of geographical variation in kernel color and carotenoid accumulation patterns across diverse production regions.

**Results:**

The regions with moderate climates (effective accumulated temperature: 1602–1694 °C; precipitation: 373–404 mm) and fertile soils produced higher carotenoid content and more yellow kernel. In contrast, two regions were delineated as thermally constrained zones, exhibiting non-optimal growing conditions with effective accumulated temperatures and diurnal temperature fluctuation. The key environmental drivers of quality variation included effective accumulated temperature, precipitation, soil total phosphorus, and organic matter, though cultivar-specific sensitivity to these factors varied significantly. Variance component analysis demonstrated that genotype was the predominant source of variation, explaining 53.39–67.71% of the total variance, while the G×E interaction accounted for 16.51–26.40%. Furthermore, GGE (Genotype plus Genotype-by-Environment Interaction) biplot analysis revealed distinct genotype-environment interactions: high-performing cultivars in terms of millet quality but less stable with varied locations were optimal for premium regions with relatively higher quality indicators, while environmentally resilient cultivars proved more suitable for marginal regions with relatively lower quality indicators. By Ultra-Performance Liquid Chromatography-Tandem Mass Spectrometry method, we detected 39 carotenoid metabolites in foxtail millet kernels, showing significant regional variations, and a geographically distinctive biomarker (zeaxanthin-myristate-palmitate) was identified.

**Conclusion:**

The findings highlight the importance of matching cultivars to regional ecological conditions to optimize foxtail millet quality. These findings provided theoretical foundations for optimizing regional cultivation strategies and improving foxtail millet quality under G×E interactions.

**Supplementary Information:**

The online version contains supplementary material available at 10.1186/s12870-025-08008-1.

## Background

Foxtail millet (*Setaria italica* L.) is a minor cereal crop widely cultivated in northern China. As a drought-resistant C_4_ crop, it exhibits remarkable tolerance to barren soils and exceptional environmental adaptability, making it a crucial crop in arid and semi-arid regions [1–4]. With increasing consumer demand for nutritious and healthy foods in recent years, foxtail millet regained attention due to its high nutritional value [5]. While recognized as a valuable source of macronutrients (carbohydrates, proteins, and fats) and dietary fiber, foxtail millet is particularly distinguished by its abundant bioactive compounds, particularly carotenoids and flavonoids. These compounds enhance its potential as a functional food composition [6–8].

The millet color and carotenoid content are important indicators for evaluating foxtail millet quality, which directly affect its commercial and nutritional value. Upon dehulling, foxtail millet grains exhibit a range of colors, of which varieties with yellow kernels account for over 90% [9]. As a class of fat-soluble pigments, carotenoids are not only critical factors in determining millet color but also confer antioxidant, anti-inflammatory, and immunomodulatory benefits [10, 11]. Notably, metabolomic analyses revealed that high-quality foxtail millet varieties exhibit similarities in carotenoid and flavonoid metabolites [12], implying a potential correlation between carotenoid components and sensory quality. At the molecular level, carotenoid accumulation was coordinately controlled by biosynthesis and degradation pathways, with key genes (e.g., *PSY*, *CCD*) playing pivotal roles in modulating pigment deposition and color development [6, 13–16].

The millet color and carotenoid content of foxtail millet were mainly influenced by production environment, genotype, cultivation practices, and processing methods [17–19]. Genetic studies demonstrated that genotype plays a decisive role in determining carotenoid content, with reported carotenoid content ranging from 5.40 to 19.55 mg/kg [1]. This genetic variation is particularly evident in Chinese summer-sown cultivars, which exhibit a wide range of b values (yellowness intensity of millet) from 54.50 to 75.35 [20]. Environmental factors also exert important influences on quality development. Meteorological factors, including growth season temperature regimes, precipitation patterns, and solar radiation intensity, collectively regulate carotenoid accumulation through both direct metabolic and indirect physiological pathways [9, 21]. Light and temperature mediate carotenoid biosynthesis by regulating the expression of key genes such as *PSY* and *DXS* [22–24]. The quality of foxtail millet was also affected by soil characteristics through their comprehensive regulation of carotenoid metabolism. Distinct soil properties, including texture, fertility levels, and pH values across various ecoregions, exerted significant influences on both the biosynthetic pathways and final accumulation patterns of carotenoids in the crop [25, 26]. In addition, the rhizosphere microbiome influences nutrient cycling and plant hormone production, indirectly affecting secondary metabolite accumulation [27].

Shanxi Province stands as one of major foxtail millet production regions in China, where its complex topography and diverse climatic conditions create distinct agroecological environments that profoundly influence growth and quality of foxtail millet [28, 29]. Notably, key environmental factors, such as temperature, precipitation, solar radiation, and soil nutrients, vary significantly across regions. These variations lead to distinct differences in grain color and carotenoid composition. Furthermore, the genotype-by-environment (G×E) interaction effects significantly influence crop quality formation [30, 31]. Although previous studies investigated individual factors influencing foxtail millet quality, systematic research on the variation characteristics of millet color and carotenoid content across multiple production regions and diverse genotypes remains insufficient. Particularly regarding G×E interactions, previous research mostly focused on limited environments or a narrow range of varieties, making it difficult to reveal the formation mechanisms of foxtail millet quality under different ecological conditions. In this research, 12 predominant foxtail millet cultivars were cultivated in 10 distinct regions of Shanxi Province in 2022 and 2023. Through integrated analysis of meteorological factors and soil chemical properties, we systematically examined the variation patterns of color parameter (yellowness intensity) and carotenoid profiles, and their relationships with ecological factors. The main objectives of this study were: (1) to elucidate the variation patterns of color parameter and carotenoid content among different production regions and cultivars; (2) to investigate genotype × environment (G×E) interactions on foxtail millet quality; (3) to investigate the relationships between color/carotenoid content and ecological factors; and (4) to characterize region-specific carotenoid profiles and identify functionally valuable components. This study will provide scientific evidence for improving foxtail millet quality and optimizing cultivar selection and cultivation zoning.

## Methods

### Experimental materials

The experiment was conducted in 2022 and 2023. We cultivated 12 major foxtail millet cultivars in 10 major regions of Shanxi Province. Detailed information regarding the cultivars and trial locations is provided in Tables S1 and S2.

The seeds of the all foxtail millet cultivars used in this study were obtained from various agricultural research institutions and breeding centers across China, as detailed in Table S1. These cultivars are publicly accessible and were supplied strictly for research purposes, with no commercial restrictions applied. No wild-collected plant materials were used in this study. Formal cultivar identification was performed by the corresponding authors based on distinct morphological and agronomic characteristics. Voucher specimens for each cultivar have been deposited in the Herbarium of the College of Agronomy, Shanxi Agricultural University, with corresponding accession numbers provided in Table S1. This study adhered to all relevant institutional and national guidelines governing the use of plant material in experimental research. As all materials consisted of commonly cultivated varieties that are neither endangered nor protected, no specific permits were required for their use.

A standardized cultivation and management protocol was uniformly implemented across all the test regions. The experiment was arranged in a randomized complete block design with three replications. Each cultivar was randomly assigned to plots within each block, with a row spacing of 30 cm and row length of 2 m. The fertilizer application rates were 150 kg/ha of N, 90 kg/ha of P₂O₅, and 75 kg/ha of K₂O. After maturation, 10 foxtail millet panicles were collected from each plot. The panicles were threshed and milled, then stored separately in 4 °C refrigerator and − 80 °C freezer for subsequent analysis.

### Meteorological data

The meteorological data were obtained from the China Meteorological Data Network (http://data.cma.cn), including the following indicators during the growth period of millet in various production regions: daily maximum temperature (HADT), daily minimum temperature (LADT), daily average temperature (ADT), precipitation (PRE), air relative humidity (ARH), diurnal temperature range (DTR), and sunshine duration (SSD).

The calculation method for effective accumulated temperature (EAT) is as follows: $\mathrm{EAT}\;=\;{\textstyle\sum_{}}\left(\mathrm{ADT}-{\mathrm T}_{\mathrm{base}}\right)$, ADT is the daily average temperature (°C), and T_base_ is the biological lower threshold temperature for millet (10 °C), only ADT ≥ 10 °C are accumulated for effective temperature (unit: °C·d).

### Soil sample collection and chemical analysis

Prior to sowing, surface soil samples (0–20 cm depth) were collected from the experimental field using a soil auger following a double-diagonal five-point sampling pattern. The five soil samples were thoroughly mixed into a composite sample, then air-dried, ground, and sieved through a 1 mm mesh for subsequent analysis. The measured soil properties included: pH, available nitrogen (AN), available phosphorus (AP), available potassium (AK), soil organic matter (SOM), total nitrogen (TN), total phosphorus (TP), available calcium (Ca), available magnesium (Mg), available iron (Fe), available boron (B). Analytical methods and standards: pH was measured by potentiometric method [32]; TP was measured by acid digestion-ICP-OES method [33]; AP was measured by Sodium bicarbonate extraction-molybdenum antimony colorimetry method [34]; AK was measured by ammonium acetate extraction-flame photometry method [35]; TN was measured by Kjeldahl method [36]; AN was measured by alkali diffusion method [37]; SOM was measured by potassium dichromate oxidation-volumetric method [38]; Fe was measured by DTPA extraction-ICP-OES method [39]; B was measured by hot water extraction-ICP-OES method [40].

### Measurement of color parameters

Using a non-contact spectrophotometer (X-Rite VS450, USA), b value of grains was measured, where + b values indicate increased yellowness while -b values indicate enhanced blueness. The detailed procedure was conducted as follows: 3 g dehulled foxtail millet grains were evenly distributed in a specialized measurement chamber for scanning to measure the b value of kernels (BVK). Subsequently, the grains were ground into powder using a planetary ball mill under liquid nitrogen freezing conditions (-196 °C, 2 min) to measure b value of powder (BVP).

### Measurement of carotenoid content

According to method of the American Association of Cereal Chemists (AACC, 2000) [41], water saturated n-butanol was used to extract carotenoid. The detailed procedure was conducted as follows: (1) Extraction solvent preparation: n-Butanol and deionized water were mixed at ratio of 1:1 (v/v), shake thoroughly then stand overnight at 4 °C for phase separation. The upper water-saturated n-butanol layer was collected for subsequent use. (2) Sample treatment: foxtail millet powder was freeze-dried using CHRIST freeze dryer (Germany) at -30 °C and 37 Pa for 48 h. (3) Extraction and centrifugation: 0.6 g of freeze-dried powder was added into a 10 mL centrifuge tube, add 6 mL water-saturated n-butanol, vortex to mix thoroughly, and place on a shaker at 200 rpm for 3 h. Subsequently, centrifuge at 4 °C and 8000×g for 15 min, and collect the supernatant as the test solution. (4) Absorbance measurement: the absorbance of the resulting supernatant was determined (three times for each sample) at 450 nm (A450) using water-saturated n-butanol as the reference. The total carotenoid content (mg/kg) was calculated using the following formula: $\left[\left(\mathrm A-{\mathrm A}_0\right)\times\mathrm V\right]/\left(0.250\;\times\mathrm m\right)$, where A is the absorbance of the test solution, A₀ is the absorbance of the blank reference, V is the extraction volume (6 mL), m is the sample mass (0.6 g).

### Carotenoid metabolomic analysis

#### Sample preparation

The sample was freeze-dried, ground into powder (30 Hz, 1.5 min), and stored at -80 °C. 50 mg powder was weighted and extracted with 0.5 mL mixed solution of n-hexane: acetone: ethanol (1:1:1, v/v/v). The extract was vortexed for 20 min at room temperature. The supernatants were collected after centrifuged at 12,000 r/min for 5 min at 4 °C. The residue was re-extracted by repeating the above steps again under the same conditions. And then evaporated to dryness, reconstituted in mixed solution of MeOH/MTBE (1:1, v/v). The solution was filtered through a 0.22 μm membrane filter for further LC-MS/MS analysis [42–44].

#### Metabolite quantification

Metabolite identification was confirmed by matching the retention time and MRM transitions against a self-built database (MWDB) established with authentic chemical standards. For semi-quantification, calibration curves for each carotenoid were constructed using a dilution series of pure reference standards across a concentration range of 0.001 to 400 µg/mL. The resulting concentrations are reported as semi-quantitative values, which are robust for comparative analysis across samples but are not absolute concentrations due to the lack of isotope-labeled internal standards for every compound.

#### Quality control

A comprehensive quality control (QC) procedure was implemented to ensure data reliability. A pooled QC sample, created from all experimental extracts, was analyzed after every 10 experimental samples to monitor instrument stability throughout the sequence. Furthermore, a mixture of authentic internal standards was spiked into each sample prior to extraction to correct for variations. Metabolites with a relative standard deviation (RSD) greater than 30% in the QC samples were excluded from subsequent data analysis.

#### UPLC conditions

The sample extracts were analyzed using an UPLC-APCI-MS/MS system (UPLC, ExionLC™ AD; MS, Applied Biosystems 6500 Triple Quadrupole). The analytical conditions were as follow, LC: column, YMC C30(3 μm, 100 mm×2.0 mm i.d); solvent system, methanol: acetonitrile (1:3, v/v) with 0.01% BHT and 0.1% formic acid (A), methyl tert-butyl ether with 0.01% BHT (B); gradient program, started at 0% B (0–3 min), increased to 70% B (3–5 min), then increased to 95% B (5–9 min), finally ramped back to 0% B (10–11 min); flow rate, 0.8 mL/min; temperature, 28 °C; injection volume: 2 µL.

#### APCI-MS/MS conditions

Linear ion trap (LIT) and triple quadrupole (QQQ) scans were acquired on a triple quadrupole-linear ion trap mass spectrometer (QTRAP), QTRAP^®^ 6500 + LC-MS/MS System, equipped with an APCI Heated Nebulizer, operating in positive ion mode and controlled by Analyst 1.6.3 software (Sciex). The APCI source operation parameters were as follows: ion source, APCI+; source temperature 350 °C; curtain gas (CUR) were set at 25.0 psi. Carotenoids were analyzed using scheduled multiple reaction monitoring (MRM). Data acquisitions were performed using Analyst 1.6.3 software (Sciex). Multiquant 3.0.3 software (Sciex) was used to quantify all metabolites. Mass spectrometer parameters including the declustering potentials (DP) and collision energies (CE) for individual MRM transitions were done with further DP and CE optimization. A specific set of MRM transitions were monitored for each period according to the metabolites eluted within this period [43, 45].

#### Differential metabolites screening

Orthogonal partial least squares-discriminant analysis (OPLS-DA) was employed to conduct intergroup differential analysis [46]. Differential metabolites were screened using dual thresholds: variable importance in projection (VIP) ≥ 1 and fold change (FC) ≥ 2 or ≤ 0.5. 

### Statistical analysis

The experimental data were recorded and processed using Microsoft Excel 2020. Genotype plus Genotype-by-Environment Interaction (GGE) biplots were generated using Genstat 21.0. Analysis of variance (ANOVA) was conducted with SPSS 18.0. Multivariate analyses including K-means clustering (flexclust package), OPLS-DA (ropls package), RDA (vegan package), and heatmap visualization (pheatmap package) were performed in R 3.6.1. Figures were created using OriginPro 2021 (OriginLab Corp., USA).

Analysis of Variance (ANOVA): A two-way analysis of variance (ANOVA) was employed to assess the effects of genotype, environment, and their interaction on foxtail millet color parameters (BVK, BVP) and carotenoid content. Prior to ANOVA, the underlying assumptions were verified. The normality of the residuals was confirmed using the Shapiro-Wilk test (*P* > 0.05), and the homogeneity of variances was assessed using Levene’s test (*P* > 0.05). No severe violations were observed. The following fixed effects model was constructed: Yij = µ + Gi + Ej + (G × E)ij + εij; where Yij represents the mean phenotypic value of the i-th genotype in the j-th environment; µ is the overall mean; Gi is the fixed effect of the i-th genotype (i = 1, 2, …, 12); Ej is the fixed effect of the j-th environment (j = 1, 2, …, 10); εij is the random error term. For significant main effects (*P* < 0.05), post-hoc pairwise comparisons were performed using Tukey’s Honest Significant Difference (HSD) test. Variance components were estimated to quantify the relative contributions of genotype, environment, and their interaction to the total phenotypic variation. The “environment” factor in this part was defined by ten specific geographical locations, the phenotypic value for each environment (location) was calculated as the mean of data from the 2022 and 2023 growing seasons.

#### GGE biplot analysis

The model was fitted via singular value decomposition (SVD) of the environment-centered data. The stability of the biplot interpretation was supported by the high total variance explained by the first two principal components (PC1 and PC2). Furthermore, the discriminativeness of test environments and the stability of genotypes, as inferred from the biplot, were validated by their consistent patterns with the ANOVA results and their robust performance across the biplot’s stability measures. (1) Adaptation GGE Biplot [47]: based on genotype-by-environment interaction effects, test sites were classified into distinct groups. A polygon was formed by connecting the most distant genotypes from the origin, and perpendicular lines were drawn from the origin to each polygon side, dividing the biplot into multiple sectors. This method identified the optimally adapted varieties within each sector. (2) Stability GGE Biplot: the Average Environment Axis (AEA) represented trait performance levels. The Average Environment Coordinate (AEC) indicated stability. Genotypes closer to the AEA exhibited higher stability. Environmental vector length reflected the discriminative power of test sites. Angles between vectors represented the representativeness of test environments.

#### Redundancy analysis (RDA)

RDA was conducted to assess the relationships between ecological factors and measured indicators. The arrows represent environmental factors, with their lengths indicating the magnitude of each factor’s influence, while the angles between arrows denote the strength and direction of correlations-acute angles (< 90°) signify positive correlations and obtuse angles (> 90°) indicate negative correlations.

## Results

### Geographic and genotypic variability in color parameters and carotenoid content​

**Fig. 1:**
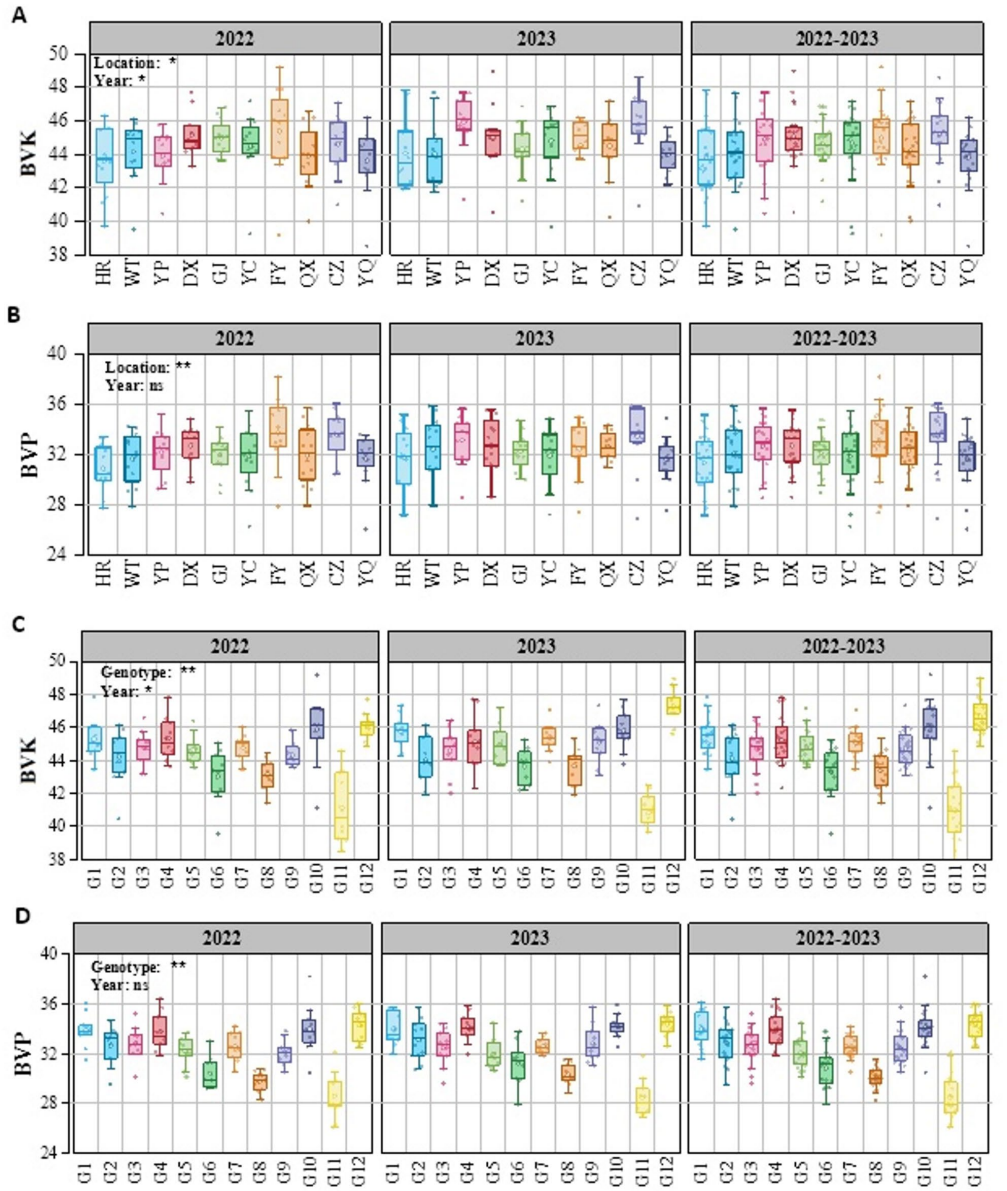
Regional variation of BVK (**A**) and BVP (**B**) of foxtail millet in 10 regions. C and D: Distribution characteristics of BVK (**C**) and BVP (**D**) among 12 foxtail millet cultivars. * and **: significant difference at 0.05 and 0.01, respectively; ns: not significant. BVK: b value of kernel; BVP: b value of powder

**Fig. 2:**
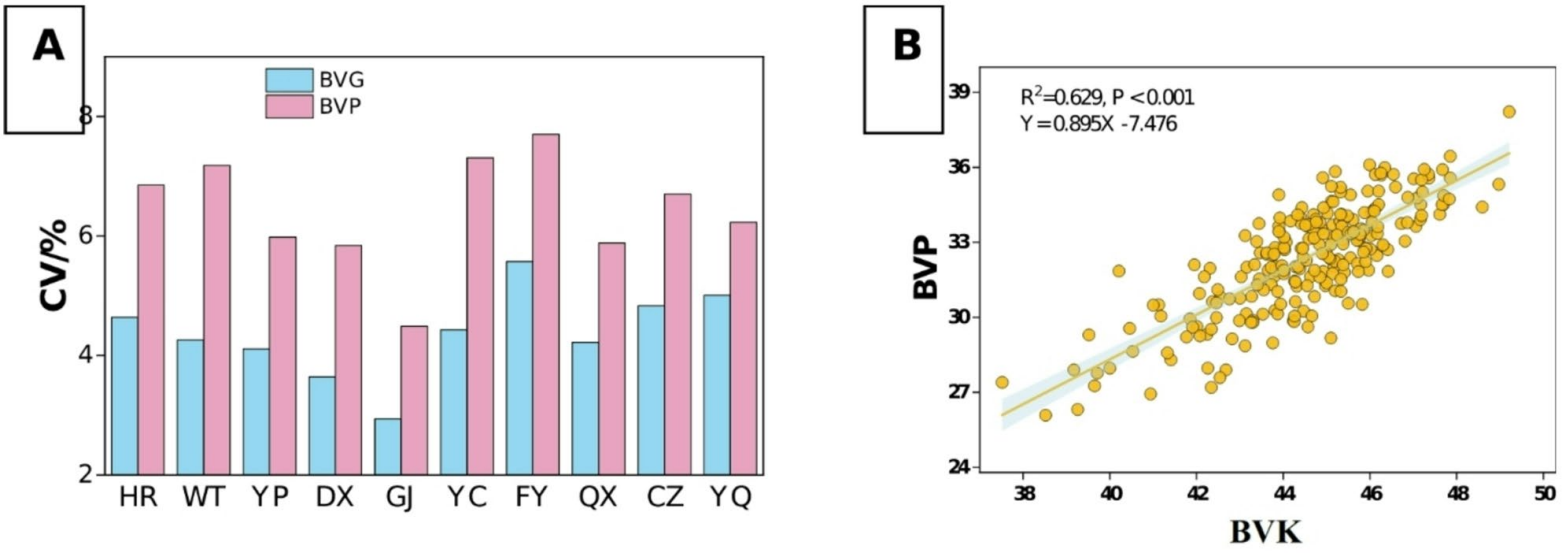
**A**: Variation degree (CV) of BVK and BVP across different regions. **B**: Variation degree (CV) of BVK and BVP among different cultivars. **C**: Correlation analysis between BVK and BVP. BVK: b value of kernel; BVP: b value of powder

Significant regional variations in BVK (b value of kernels) were observed among different regions (Fig. 1-A, *P* < 0.05). In 2022, FY, DX, and GJ exhibited higher BVK, exceeding the overall mean (44.38) by 1.40%-2.25%, whereas HR (43.59) and YQ (43.74) exhibited lower values. Moreover, significant interannual variation was observed (*P* < 0.05). In 2023, the highest BVK values were observed in YP (45.82) and CZ (45.80), surpassing the overall mean (44.68) by 2.50%, while northern (HR: 44.06; WT: 44.01) and southern (YQ: 43.96) regions maintained lower values. Notably, CZ and FY consistently maintained higher BVK across both years, demonstrating stable regional superiority. Variability analysis (CV) revealed FY and YQ exhibited greater variation, while GJ and CZ demonstrated more stable performance (Fig. 2-A). Cultivar differences in BVK were also significant (Fig. 1-C, *P* < 0.01). G1, G10, and G12 consistently exhibited higher BVK, which were 2.81%-4.83% higher than the overall mean (44.53). In contrast, G6 (43.32), G8 (43.40), and G11 (40.98) consistently exhibited lower values.Variability analysis (CV) identified G10 (3.79%) and G11 (4.37%) were higher variability cultivars, and G5 (2.06%) and G7 (1.99%) were relatively stable cultivars (Fig. S1-A).

We also observed significant regional variations in BVP (b value of powder; Fig. 1-B, *P* < 0.05). In 2022, FY (33.78) and CZ (33.60) showed higher BVP, exceeding the overall mean (32.26) by 4.71% and 4.15%, respectively; while HR (30.93) and WT (31.68) exhibited lower BVP. The overall BVP in 2023 were slightly higher than those in 2022. CZ and YP exhibited relatively higher BVP, which were 2.83% and 2.15% higher than the overall mean (32.49), respectively, while YQ and HR showed relatively lower BVP. CZ and FY showed higher BVP performance among all regions in two years, whereas HR and YQ consistently ranked lowest. Variability analysis (CV) revealed that FY (7.70%) and YC (7.31%) were high-variation regions, while GJ and DX were low-variation regions (Fig. 2-A). For cultivars, G1, G4, G10, and G12 consistently exhibited higher levels across both years, exceeding the two-year overall mean (32.37) by 4.53%-6.30% (Fig. 1-D). Conversely, G8 (30.04) and G11 (28.51) consistently showed lower BVP. Variability analysis (CV) revealed that G6 (5.01%) and G11 (5.90%) were high-variation cultivars, while G7 (2.78%) and G8 (2.80%) were relatively stable cultivars (Fig. S1-A). Notably, correlation analysis revealed a highly significant positive relationship between BVP and BVK (*R*² = 0.629, *P* < 0.001, Fig. 2-B).

**Fig. 3:**
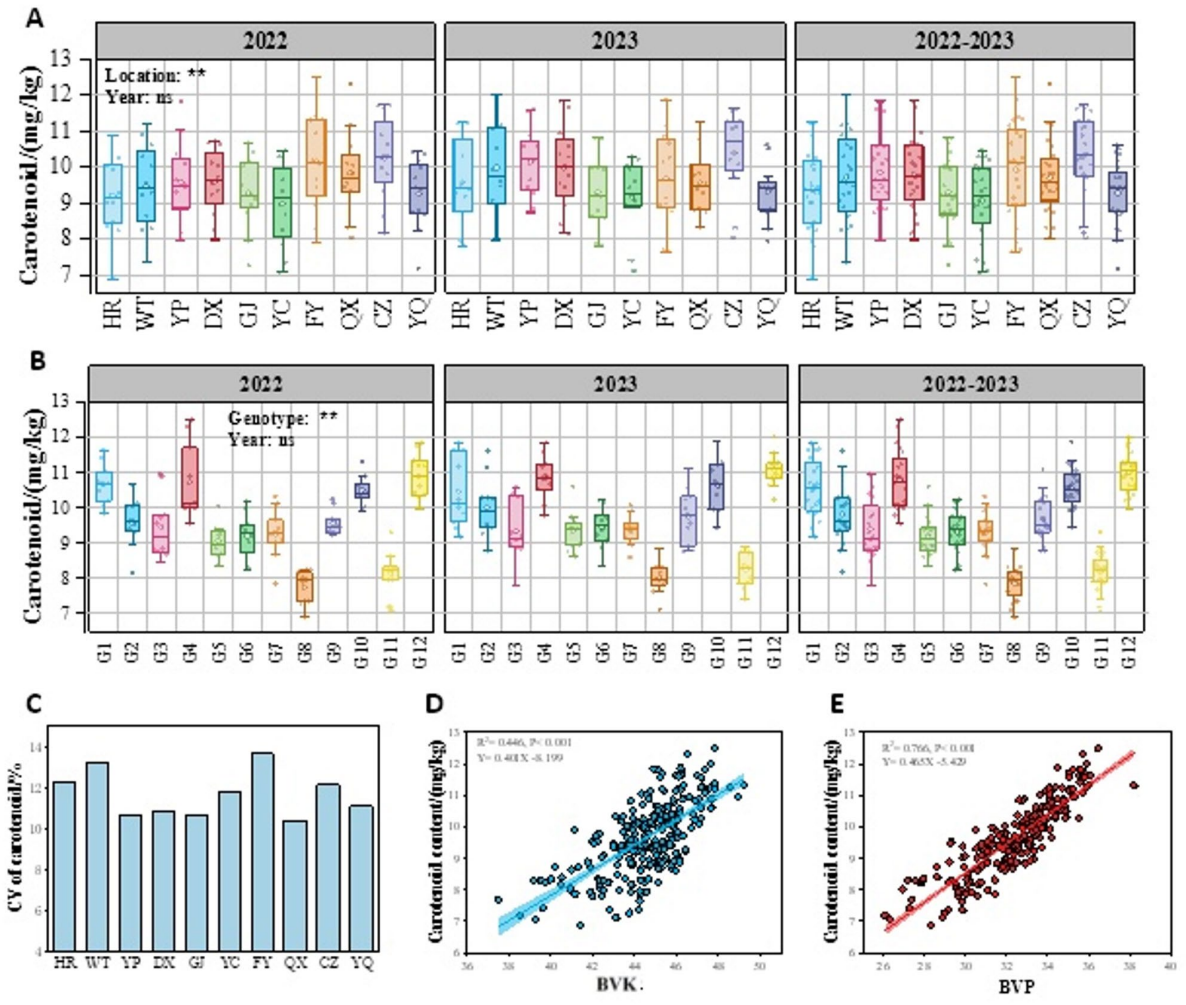
**A**: Regional variation of carotenoid content in foxtail millet in 10 ecological regions. **B**: Distribution characteristics of carotenoid content among 12 foxtail millet cultivars. **C**: Variation degree of carotenoid content in foxtail millet in different regions. **D**: Correlation analysis between BVK and carotenoid content. **E**: Correlation analysis between BVP and carotenoid content. * and **: significant difference at 0.05 and 0.01, respectively; ns: not significant. BVK: b value of kernel; BVP: b value of powder

The carotenoid contents showed significant regional variations (Fig. 3-A, *P* < 0.05). In 2022, regional mean values ranged from 8.99 to 10.26 mg/kg (overall mean: 9.56 mg/kg). CZ and FY showed the highest carotenoid contents (> 10.00 mg/kg), while HR and YC exhibited lower contents (< 9.20 mg/kg). In 2023, the overall carotenoid contents increased compared to 2022, with a range of 9.16–10.33 mg/kg. WT, YP, DX and CZ had relatively higher carotenoid contents (exceeding 10.00 mg/kg), while GJ and YC exhibited lower contents. A two-year comparative analysis identified CZ and FY as optimal production regions for carotenoid contents, whereas GJ, YC, HR and YQ were inferior regions. Variability analysis indicated that FY (13.66%) and WT (13.24%) were high-variation regions, while QX and GJ were low-variation regions (Fig. 3-C). Among cultivars, G1, G4, and G12 consistently exhibited higher levels (> 10.50 mg/kg), whereas G8 (7.87 mg/kg) and G11 (8.18 mg/kg) maintained lower values (Fig. 3-B). Variability analysis (CV) revealed that G2 (8.14%) and G3 (9.01%) were high-variation cultivars, while G1(5.57%), G5 (5.45%) and G4 (5.23%) were low-variation cultivars (Fig. S1-B).

Correlation analysis demonstrated that both BVK and BVP exhibited highly significant relationships with carotenoid contents (*P* < 0.001; Fig. 3-D&E). Notably, BVP demonstrated a stronger correlation (*R*² = 0.766) with carotenoid content than BVK.

### Genotype × environment (G×E) interaction analysis

Analysis of variance revealed a significant G×E interaction effect on carotenoid content and BVK (*P* < 0.05). Both genotype and environment (location) showed highly significant effects on all indicators (*P* < 0.001). Variance component analysis showed the tendency of genotype > G×E interaction > environment (location). Specifically, genotype effects explained 58.65% (BVK), 67.71% (BVP), and 53.39% (carotenoid content) of the total variation. Corresponding G×E interaction contributions were 24.24% (BVK), 16.51% (BVP), and 26.40% (carotenoid content) (Table S3).

**Fig. 4:**
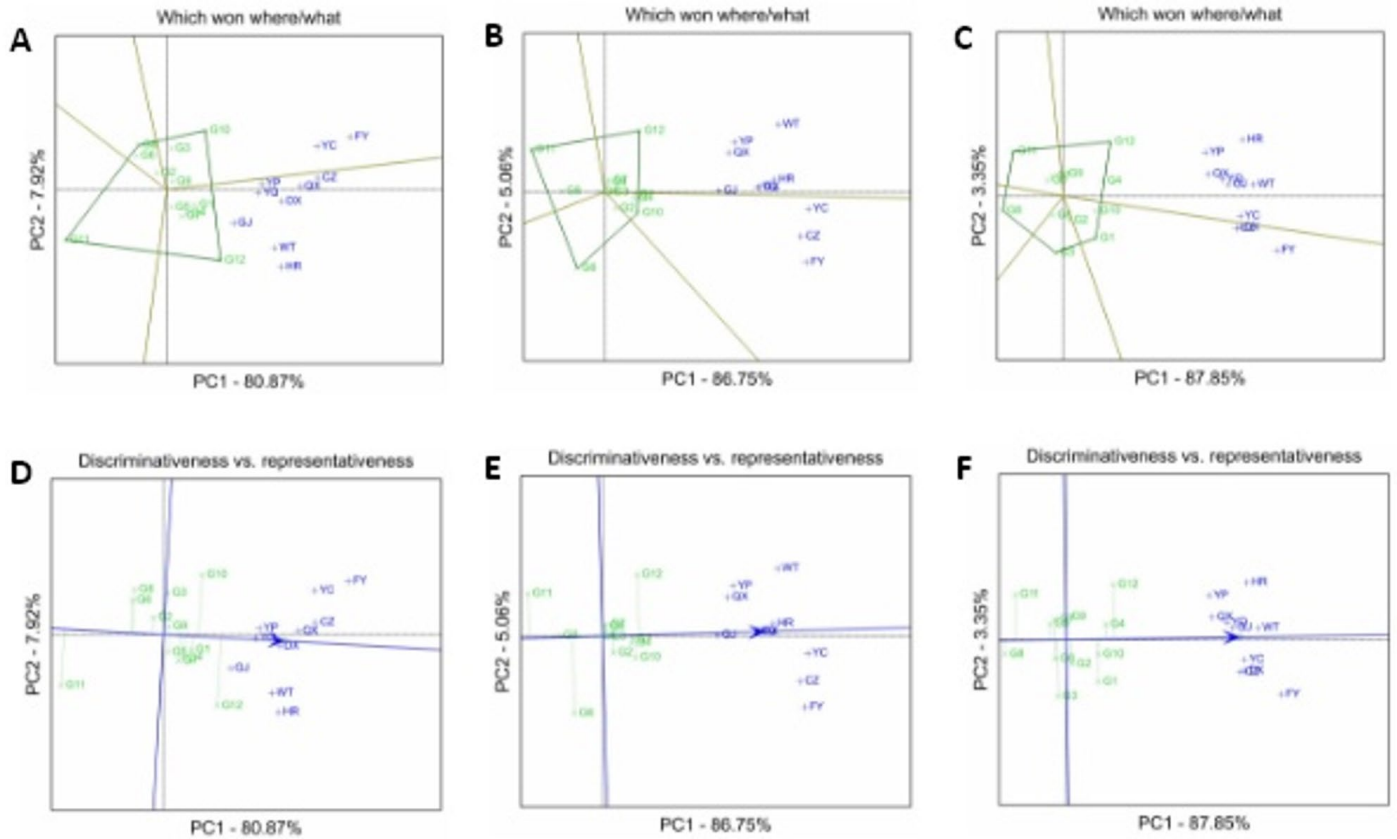
**A**-**C**: GGE biplot analysis for adaptability evaluation of BVK (**A**), BVP (**B**), and carotenoid content (**C**). **D**-**F**: GGE biplot analysis for stability and representativeness evaluation of BVK (**D**), BVP (**E**), and carotenoid content (**F**). BVK: b value of kernel; BVP: b value of powder

The GGE biplot analysis revealed that the genotype and G×E interaction effects collectively accounted for 88.79% (BVK), 91.81% (BVP), and 91.20% (carotenoid content) of the total variation. For BVK (Fig. 4-A), the polygon formed by G8, G11, G12, and G10 divided the plot into four sectors; YC and FY fell within one sector where G10 showed the highest BVK, the remaining eight sites clustered in another sector where G12 exhibited superior performance. For BVP (Fig. 4-B), the polygon formed by G6, G10, G11, and G12 divided the plot into four sectors; GJ, YC, CZ, and FY grouped in one sector, where G10 exhibited the highest BVP, the other 6 test sites formed another sector where G12 showed optimal BVP. For carotenoid content (Fig. 4-C), the polygon formed by G1, G3, G8, G11, and G12 divided the plot into five sectors; DX, CZ, and FY clustered in one sector where G12 exhibited highest carotenoid content. In the remaining sector containing 7 test sites, G1 showed relatively higher carotenoid content.

The GGE biplot stability analyses revealed distinct patters in cultivar performance stability and environmental discrimination. For BVK (Fig. 4-D), cultivars G1, G4, and G5 exhibited optimal environmental stability, while G12 displayed the poorest stability; test sites FY, YC, and HR showed superior genotype-discriminating capacity, whereas YC and YP exhibited limited discrimination. In BVP analysis (Fig. 4-E), G1, G3, and G5 maintained high stability, contrasting with the unstable performance of G6, G11, and G12; FY, WT, and CZ emerged as sites with strong discrimination capacity, while DX and GJ demonstrated minimal differentiation. For carotenoid content (Fig. 4-F), G4, G5, and G8 were identified as the most stable cultivars, whereas G3, G12, and G11 showed poorest stability; FY and HR displayed high discrimination efficiency, while QX, YC, and GJ had limited capacity to distinguish genotype.

Furthermore, analysis of the relationship between regional stability and trait performance revealed that although BVK and BVP showed negative correlations with the coefficient of variation (CV), these relationships were not statistically significant (Fig. S2-A&B), suggesting that the superiority of kernel color traits is not strongly associated with their stability. In contrast, carotenoid content showed a significant positive correlation with CV (*P* < 0.05, Fig. S2-C), indicating that cultivars with higher carotenoid content may possess better regional stability.

### Relationship between color parameters and carotenoid content with ecological factors

**Fig. 5:**
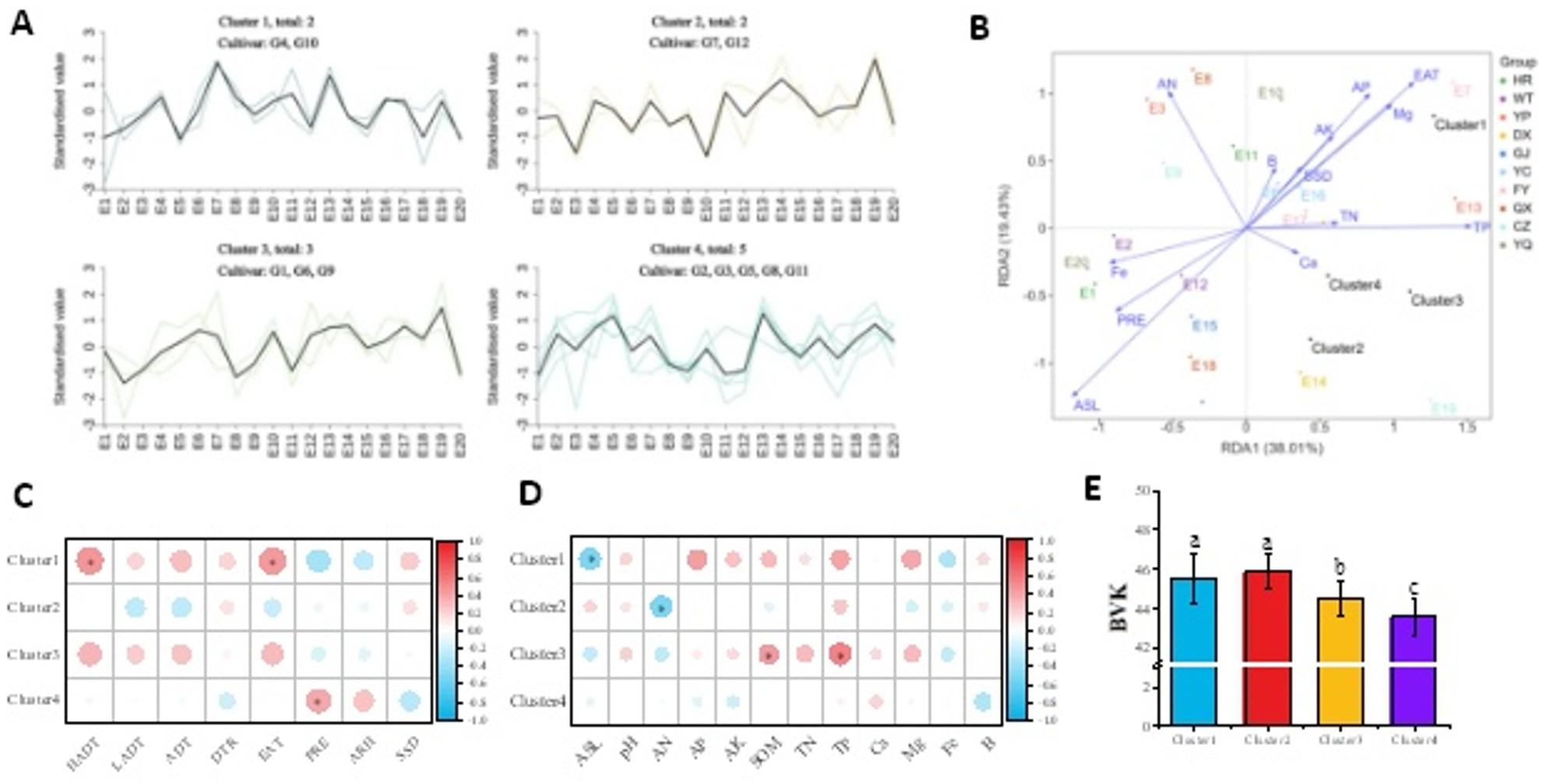
**A**: Variation trends of BVK among four foxtail millet cultivar clusters (K-means clustering method) across 20 ecological sites. **B**: RDA analysis of foxtail millet BVK and environmental factors. **C**-**D**: Correlation analysis between BVK and ecological factors for different foxtail millet clusters. **E**: Comparison of BVK among different foxtail millet clusters. * indicates significant difference at 0.05. BVK: b value of kernel; HADT: daily maximum temperature; LADT: daily minimum temperature; ADT: daily average temperature; PRE: precipitation; ARH: air relative humidity; DTR: diurnal temperature range; SSD: sunshine duration; Alt: altitude; AN: available nitrogen; AP: available phosphorus; AK: available potassium; SOM: soil organic matter; TN: total nitrogen; TP: total phosphorus; Ca: available calcium; Mg: available magnesium; Fe: available iron; B: available boron. E1-E20: 10 geographical locations studied over two growing seasons (2022 and 2023)

In this study, significant difference on meteorological and soil factor was observed in different production regions during 2022–2023 (*P* < 0.01, Table S4&S5). Based on the variation of BVK across 20 ecological environments (10 locations×2 years), 12 cultivars were divided to 4 clusters (Fig. 5-A). Cluster 1 and Cluster 2 exhibited higher BVK values, followed by Cluster 3, while Cluster 4 showed the lowest BVK values (Fig. 5-E). RDA indicated RDA1 and RDA2 collectively explained 57.44% of variability (Fig. 5-B). Meteorological factors including EAT and PRE, along with soil factors such as ASL, TP, AN, AP and Mg, exhibited relatively strong correlations with BVK. Further correlation analysis between BVK and ecological factors for each cluster were conducted (Fig. 5-C&D). Cluster 1 showed significant positive correlations with HADT and EAT (*P* < 0.05) but significant negative correlation with ASL (*P* < 0.05); Cluster 2 and Cluster 3 showed no significant correlations with meteorological factors, and Cluster 2 showed significant negative correlation with AN (*P* < 0.05) while Cluster 3 showed significant positive correlations with both SOM and TP (*P* < 0.05); Cluster 4 showed significant positive correlation with PRE (*P* < 0.05).

**Fig. 6:**
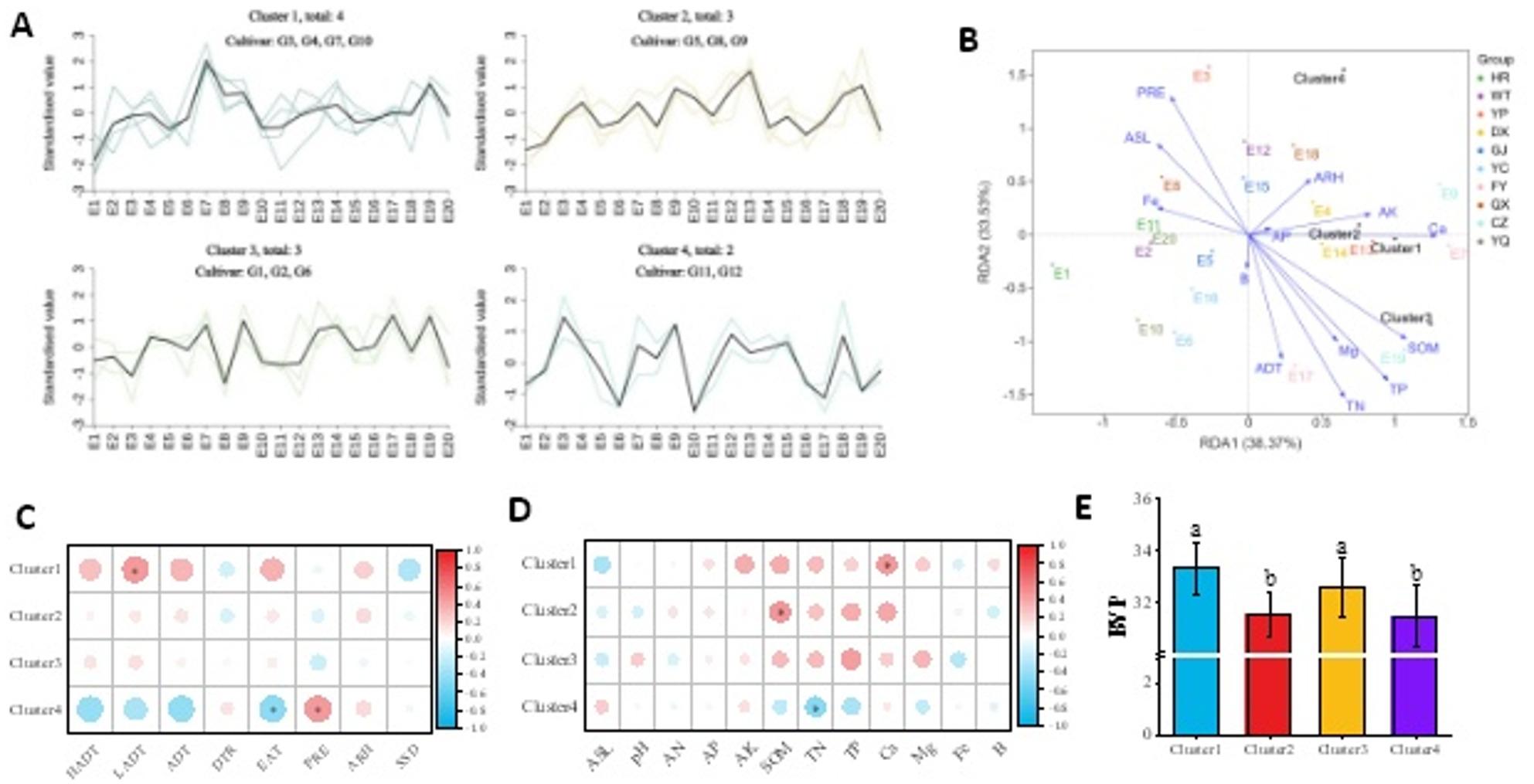
**A**: Variation trends of BVP among four foxtail millet cultivar clusters (K-means clustering method) across 20 ecological sites. **B**: RDA analysis of foxtail millet BVP and environmental factors. **C**-**D**: Correlation analysis between BVP and ecological factors for different foxtail millet clusters. **E**: Comparison of BVP among different foxtail millet clusters. * indicates significant difference at 0.05. BVP: b value of powder; HADT: daily maximum temperature; LADT: daily minimum temperature, ADT: daily average temperature; PRE: precipitation; ARH: air relative humidity; DTR: diurnal temperature range; SSD: sunshine duration; Alt: altitude; AN: available nitrogen; AP: available phosphorus; AK: available potassium; SOM: soil organic matter; TN: total nitrogen; TP: total phosphorus; Ca: available calcium; Mg: available magnesium; Fe: available iron; B: available boron. E1-E20: 10 geographical locations studied over two growing seasons (2022 and 2023)

Based on BVP variation trends, the 12 cultivars were classified into four clusters (Fig. 6-A), with Cluster 1 and Cluster 3 showing significantly higher BVP than Cluster 2 and Cluster 4 (*P* < 0.05, Fig. 6-E). RDA results indicated that RDA1 and RDA2 explained a cumulative explanatory rate of 71.90%. Meteorological factors (PRE, ADT) and soil factors (TN, TP, SOM, Ca) showed relatively strong correlations with BVP (Fig. 6-B). Correlation analysis revealed that Cluster 1 exhibited significant positive correlations with LADT and Ca (*P* < 0.05); Cluster 2 and Cluster 3 showed no significant correlations with meteorological factors, and Cluster 2 demonstrated a positive correlation with SOM (*P* < 0.05); Cluster 4 was positively correlated with PRE but negatively correlated with EAT and TN (*P* < 0.05, Fig. 6-C&D).

**Fig. 7:**
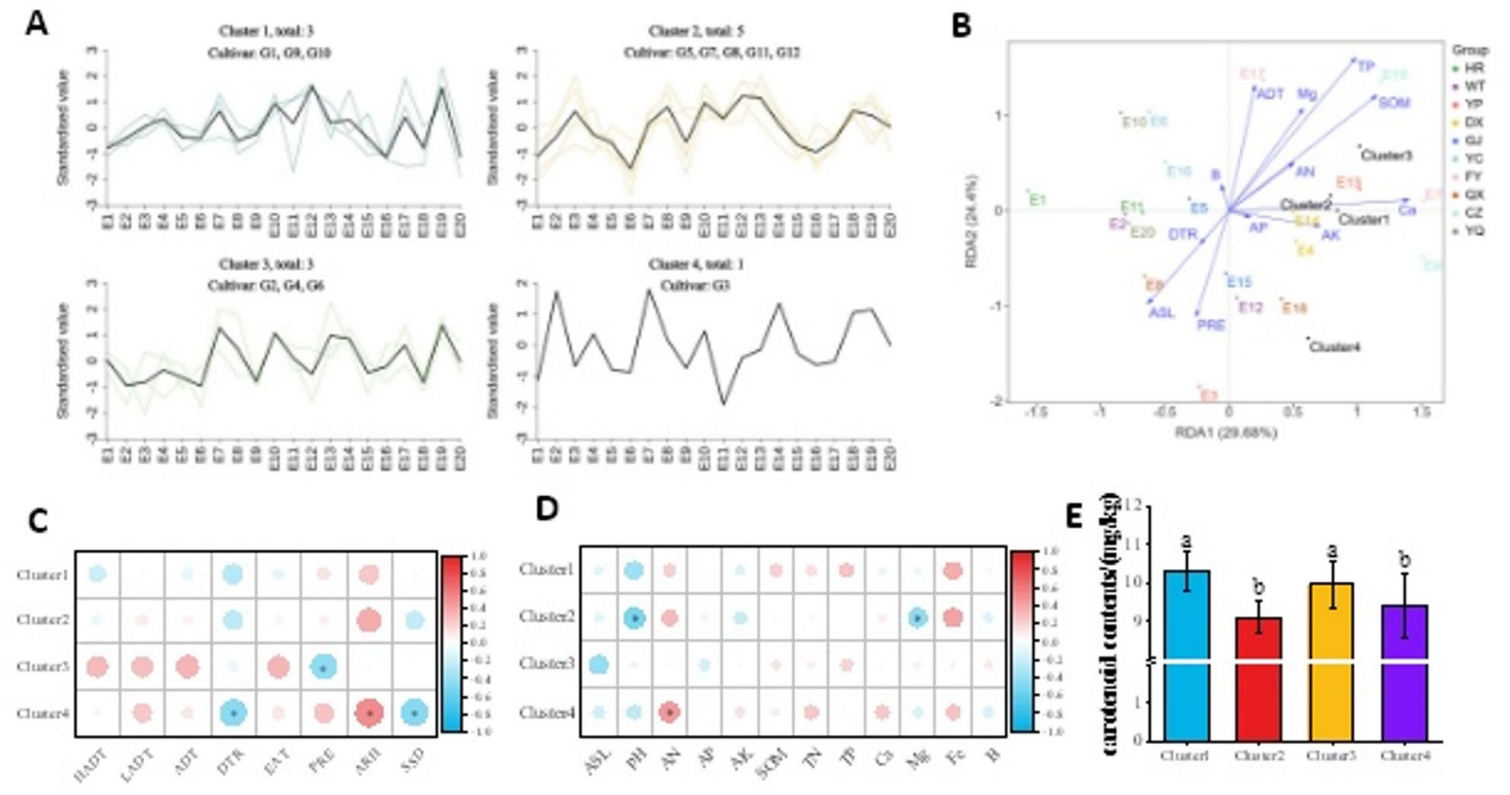
**A**: Variation trends of carotenoid content among four foxtail millet cultivar clusters (K-means clustering method) across 20 ecological sites. **B**: RDA analysis of foxtail millet carotenoid contentand environmental factors. **C**-**D**: Correlation analysis between carotenoid content and ecological factors for different foxtail millet clusters. **E**: Comparison of carotenoid content among different foxtail millet clusters. * indicates significant difference at 0.05. HADT: daily maximum temperature; LADT: daily minimum temperature, ADT: daily average temperature; PRE: precipitation; ARH: air relative humidity; DTR: diurnal temperature range; SSD: sunshine duration; Alt: altitude; AN: available nitrogen; AP: available phosphorus; AK: available potassium; SOM: soil organic matter; TN: total nitrogen; TP: total phosphorus; Ca: available calcium; Mg: available magnesium; Fe: available iron; B: available boron. E1-E20: 10 geographical locations studied over two growing seasons (2022 and 2023)

Similarly, cultivars grouped into four clusters based on carotenoid content (Fig. 7-A), with Cluster 1 and Cluster 3 showing significantly higher carotenoid content than Cluster 2 and Cluster 4 (*P* < 0.05, Fig. 7-E)). RDA results demonstrated that RDA1 and RDA2 collectively explained 54.08% of variability (Fig. 7-B). Meteorological factors (PRE, ADT) and soil factors (TP, SOM, Ca, ASL) exhibited strong correlations with carotenoid content. Further correlation analysis revealed that Cluster 1 showed no significant correlations with any ecological factors; Cluster 2 showed no significant correlations with meteorological factors but showed negative correlations with pH and Mg (*P* < 0.05); Cluster 3 showed no significant correlations with soil factors but exhibited negative correlation with PRE (*P* < 0.05); Cluster 4 (only G3) exhibited positive correlations with ARH and AN (*P* < 0.05), but negative correlations with DTR and SSD (*P* < 0.05, Fig. 7-C&D).

### Carotenoid metabolome analysis

**Fig. 8:**
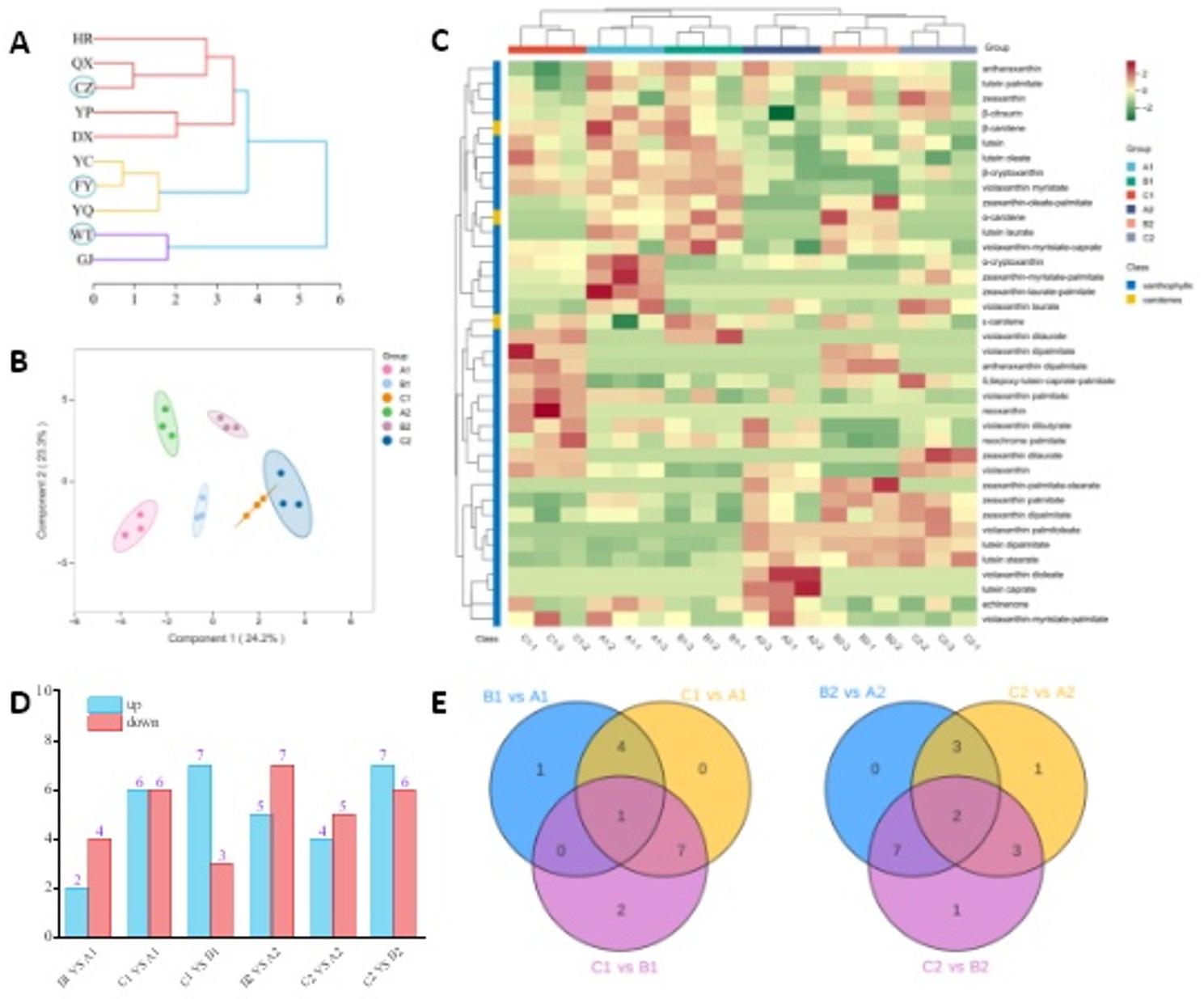
**A**: Cluster analysis of different ecological sites. **B**: OPLS-DA score plot of different samples. **C**: Heatmap of carotenoid metabolites across different samples. **D**: Comparison of differential carotenoid metabolites among sample groups. **E**: Venn diagram of differential carotenoid metabolites among sample groups. A1: WT-G1; B1: FY-G1; C1: CZ-G1; A2: WT-G3; B2: FY-G3; C2: CZ-G3

In this study, 10 production regions were classified into three groups based on meteorological variations (Fig. 8-A). Representative regions from each group - CZ (southern Shanxi), FY (central Shanxi), and WT (northern Shanxi) - were selected for metabolomic analysis using two elite cultivars (G1 and G3) that exhibit different regional adaptation patterns. These sites demonstrated significant geographical gradients in precipitation, mean daily temperature, and elevation, providing comprehensive environmental representation. From these samples, we identified 39 carotenoid metabolites, including 36 xanthophyll esters and 3 carotenes. Metabolomic profiling revealed 28 carotenoid metabolites common to all three regions (WT/FY/CZ), while region-specific signatures were identified with 33 metabolites in WT (including 5 unique), 31 in FY (3 unique), and 32 in CZ (4 unique) (Fig. 8-C), demonstrating distinct ecological impacts on carotenoid composition. OPLS-DA modeling demonstrated distinct inter-group separation with minimal intra-group variation (Fig. 8-B).

**Fig. 9:**
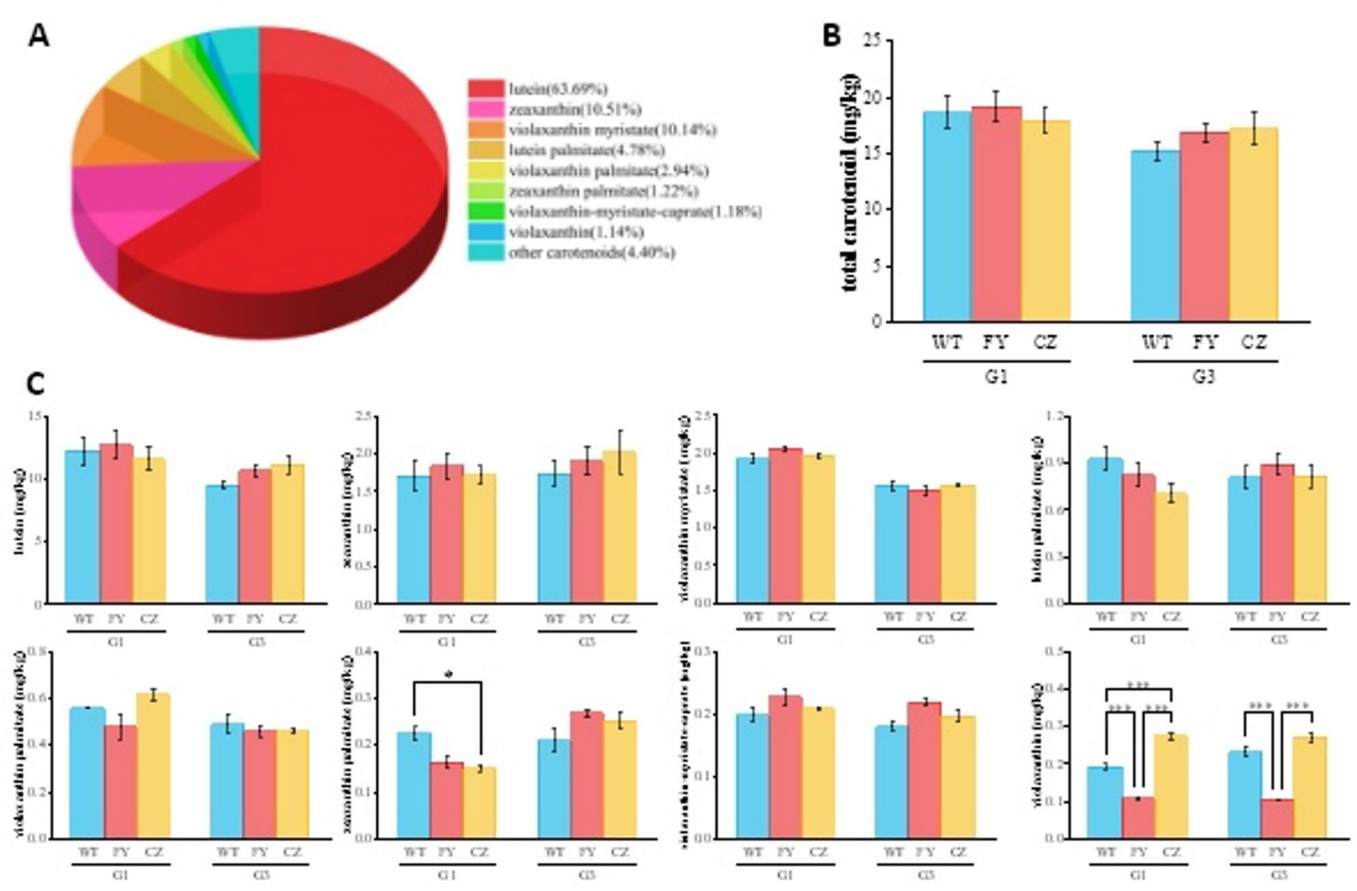
**A**: Component analysis of the identified carotenoid metabolites. **B**: Comparison of total carotenoid content across different samples. **C**: Differential analysis of major carotenoid metabolite components. A1: WT-G1; B1: FY-G1; C1: CZ-G1; A2: WT-G3; B2: FY-G3; C2: CZ-G3. * and ***: significant difference at 0.05 and 0.001, respectively

Differential metabolites were screened based on thresholds of fold change (FC ≥ 2 or FC ≤ 0.5) and variable importance in projection (VIP ≥ 1) (Table S6, Fig. 8-D&E). For cultivar G1, the FY vs. WT comparison identified 6 differential metabolites (2 upregulated, 4 downregulated); the CZ vs. WT comparison revealed 12 differential metabolites (6 upregulated, 6 downregulated); the CZ vs. FY comparison detected 10 differential metabolites (7 upregulated, 3 downregulated). Notably, zeaxanthin-myristate-palmitate was commonly identified across all three comparisons. For G3, the FY vs. WT comparison showed 12 differential metabolites (5 upregulated, 7 downregulated); the CZ vs. WT comparison demonstrated 9 differential metabolites (4 upregulated, 5 downregulated); the CZ vs. FY comparison exhibited 13 differential metabolites (7 upregulated, 6 downregulated). Two signature differential metabolites (violaxanthin dibutyrate and zeaxanthin-myristate-palmitate) were consistently found in all three comparison groups. Eight major carotenoid metabolites collectively accounted for 95.6% of the total carotenoid content (each > 1.00%, Fig. 9-A). Lutein was the most abundant (63.7%), followed by zeaxanthin (10.5%) and violaxanthin myristate (10.1%). The remaining five components accounted for 1.14%–4.78% of the total content. The total carotenoid content showed no significant variations across the three regions for both G1 and G3 cultivars (Fig. 9-B). Among the eight major carotenoid components, zeaxanthin palmitate and violaxanthin showed significant regional differences (*P* < 0.05). For zeaxanthin palmitate, G1 in the WT was significantly higher than that of CZ. For violaxanthin, both G1 and G3 cultivars displayed significantly higher contents in WT and CZ regions than in FY, with G1 in CZ being significantly higher than in WT (*P* < 0.05, Fig. 9-C). This genotype-dependent accumulation of key metabolites like zeaxanthin palmitate in specific environments constitutes a direct metabolic manifestation of genotype-by-environment (G×E) interactions.

## Discussion

### Geographic and genotypic variability in color parameters and carotenoid content of foxtail millet

This study revealed significant regional variations in color parameters and carotenoid content in foxtail millet. These geographical differences were strongly associated with Shanxi Province’s varied topography and climatic conditions [29]. The study revealed distinct climatic characteristics between the northernmost (HR) and southernmost (YQ) regions of Shanxi Province (Table S5). HR exhibited the highest DTR (12.2 °C on average during two growing seasons) coupled with the lower EAT (1383.8 °C), while YQ showed the opposite pattern - minimal DTR (8.5 °C) but the highest EAT (1817.1 °C). These extreme thermal conditions appeared to limit optimal carotenoid accumulation. In contrast, the central-southern regions (CZ and FY) exhibited more favorable climatic conditions: relatively higher EAT (CZ: 1602.4 °C; FY: 1694.8 °C) combined with balanced PRE (CZ: 373.2 mm; FY: 404.5 mm). Moreover, these regions maintained relatively superior soil fertility profiles, consistently showing higher levels of SOM, TN, TP, and Ca in both two years (Table S4). This optimal combination of climate and soil appears to create an ideal ecological condition for enhanced carotenoid biosynthesis and accumulation by regulating carotenoid biosynthesis genes (such as *PSY*, *CCD*) and hormone signals (such as ABA, strigolactones) [26, 48]. Notably, significant cultivar-specific variation in carotenoid content within these regions highlights the importance of genotype selection to fully exploit local agroecological advantages.

Significant genotypic variation was observed in both color parameters and carotenoid content. Elite cultivars (G1, G4, G10, and G12) consistently demonstrated superior performance, while G6, G8, and G11 showed markedly lower values. These findings corroborate previous reports of strong genotype-dependent correlations between millet color and carotenoid profiles, the allelic variation of *PSY1* gene was a key factor affecting the yellowness and carotenoid metabolism of foxtail millet [10, 49]. Notably, the observed BVK and carotenoid contents were higher and more stable than those reported by Yang et al. (2012) [50], likely attributable to the recent released cultivars used in our study. This further underscores the critical role of genetic factors in determining foxtail millet quality traits [9]. At the molecular level, differential expression of key biosynthetic genes (particularly *PSY* and *CCD*) among genotypes provides a mechanistic explanation for pigment accumulation patterns [6, 13]. Correlation analysis confirmed highly significant associations between color parameters and carotenoid content (*P* < 0.001), with BVP showing particularly strong predictive value (R² = 0.766, *P* < 0.001) for carotenoid levels, consistent with the findings of Ma (2023) [49]. However, cultivar-specific differences in carotenoid composition may contribute additional variation to color expression [51].

Crop quality is determined by the combined effects of genotype, environment and genotype × environment (G×E) interaction [52]‌. ‌While traditional ANOVA can detect G×E interaction, it fails to evaluate the discriminability and representativeness of test sites. In contrast, the GGE model accounts for both G and G×E effects, enabling simultaneous evaluation of genotypes and test environments [47, 53]. Therefore, this study combines ANOVA and GGE models to systematically analyze G×E interaction effects‌. ANOVA analysis revealed that genotype contributions of BVK, BVP, and carotenoid content reached 58.65%, 67.71%, and 53.39%, respectively, significantly surpassing the effects of environmental factors and G×E interactions. These findings corroborate previous reports emphasizing the crucial role of genetic factors in quality determination, highlighting the need for further exploration of superior genetic resources and in-depth investigation of carotenoid metabolic pathway genes [54]. Notably, the G×E interaction exhibited a significant effect on BVK‌, indicating the importance of environmental conditions in kernel color.

The GGE biplot analysis provided further insights into cultivar adaptability and stability. Among the four high-quality cultivars (G1, G4, G10, G12), G12 and G10 exhibited superior performance in most regions but lower stability, whereas G1 and G4 demonstrated greater stability but moderate performance. The analysis also indicated that high-performing but variable cultivar was more suitable for relatively superior production regions, whereas stable cultivars performed optimally in marginal environments. These results offered references for precious cultivar deployment strategies [52]. Regarding test site evaluation, ideal core trial locations should combine strong discriminatory power with representativeness of target environments [47]. In this study, FY and HR can be ideal core trial locations for strong discrimination, while YC and YP exhibited weaker discrimination. These findings provided scientific guidance for optimizing regional trial networks.

### Ecological factors affecting color parameters and carotenoid content

Previous studies have reported inconsistent findings regarding the relationship between meteorological factors and foxtail millet quality. Ma et al. (2023) reported that mean temperature and rainfall were positively correlated with BVK and carotenoid content [21], whereas Ning et al. (2017) found that excessive rainfall reduced lutein stability [55]. Yang et al. (2019) further identified that moderate growing degree days combined with lower precipitation promoted carotenoid accumulation [56]. However, these studies were limited by their restricted geographical coverage, preventing a comprehensive understanding of precipitation’s dual effects. Optimal soil moisture enhances root nutrient acquisition, thereby providing precursors and energy for carotenoid biosynthesis [25]. In contrast, excessive precipitation reduces photosynthetic active radiation and subsequently downregulates key carotenoid biosynthetic genes including *PSY1* and *DXS1* through photoreceptor-mediated signaling pathways [22]. As for thermal effects, carotenoid biosynthesis is mediated via temperature-sensitive modulation of the MEP pathway, particularly through ClpB3-dependent stabilization of the rate-limiting DXS enzyme [24]. Furthermore, Yungyuen et al. (2018) demonstrated that within the optimal temperature range (10–20 °C), the key carotenoid biosynthesis genes in citrus, including *CitPSY*, *CitPDS*, and *CitZDS*, were significantly upregulated; while temperatures beyond this threshold showed minimal effects on transcriptional activity [57]. This finding aligned with both our current results and those of Yang et al. (2019), confirming that moderate climatic conditions are most conducive for carotenoid accumulation in foxtail millet [56]. However, it should be noted that previous investigations were also limited by narrow germplasm representation, which constrained their ability to fully characterize genotype-specific environmental responses.

Through a multi-cultivar and multi-environment systematic analysis, this study revealed: (1) Temperature and precipitation are key meteorological drivers, but cultivar responses exhibited significant divergence; distinct cultivar clusters were identified based on their sensitivity to PRE and EAT; a cluster of cultivars showed a significant negative correlation between carotenoid content and PRE. (2) SSD was overall negatively correlated with carotenoid content possibly due to strong light suppressing *PSY1* gene expression [23]. (3) BVK and BVP were more sensitive to meteorological factors than carotenoid content. Based on these findings, we recommend implementing precision cultivar allocation according to precipitation and thermal patterns during growing-period, which provides a scientific basis for advancing smart agriculture development. It is important to note that while sunshine duration was analyzed, the current study lacked quantitative data on light intensity and ultraviolet (UV) radiation due to constraints in the available meteorological datasets. These factors are known to influence carotenoid biosynthesis pathways, for instance, by modulating the expression of genes such as *PSY* and *CCD*. The negative correlation observed with sunshine duration may hint at such complex regulatory mechanisms.

Compared with meteorological factors, cultivar types showed more consistent responses in color and carotenoid content to soil factors. SOM and TP showed positive correlations with both BVK and carotenoid contents, which was similar to previous findings. Soil phosphorus directly contributes to pigment biosynthesis by enhancing key enzymes activity for carotenoid synthesis (Cazzonelli et al., 2010) [58]. Meanwhile, SOM may improve carotenoid stability by promoting esterification [59, 60]. Additionally, the significant positive correlation between BVP and Ca was observed for a cluster of cultivars, likely attributable to calcium’s involvement in stabilizing cell wall architecture, consequently affecting pigment presentation [61]. Conversely, carotenoid content showed a negative correlation with soil pH, possibly due to pH-sensitive activity of key biosynthetic enzymes—consistent with findings by Niaz et al. (2023) [26]. In low-phosphorus soils, optimized phosphorus fertilization or organic amendments to boost soil organic matter could effectively increase both nutritional and commercial quality of foxtail millet.

Recent advances in Genomic Prediction (GP) and Genotype-by-Environment Interaction (GEI) studies have introduced innovative approaches to decipher environmental response mechanisms in crop traits [62, 63]. Lopez et al. (2015) innovatively proposed an environment-specific marker effect decomposition model, distinguishing between environment-independent main effects (stability components) and environment-specific effects (interaction components) [64]. This framework aligns with our observations of differential responses of foxtail millet color indicators to meteorological variables. Monteverde et al. (2019) incorporated multidimensional environmental covariates into GP models to predict quality traits in untested environments [65]. However, specific prediction models of foxtail millet remain underdeveloped. Future research should focus on the interactive regulatory networks between molecular markers and environmental factors to enhance predictive accuracy.

### Region-specific characteristics of carotenoid metabolomes

UPLC-MS/MS-based metabolomic analysis revealed geographical variations in carotenoid composition across different regions. The study identified 39 carotenoid metabolites across all samples, with lutein constituting the dominant component (63.7% of total content). While the prevalence of lutein is consistent with reports in other crops [12], foxtail millet is distinguished by its characteristically high abundance and diversity of xanthophyll esters. This diversity exceeded the 22 metabolites reported by Ma (2023) in two varieties (JG21 and B99) from a single production area [49], highlighting the combined effects of environmental and genotypic factors on carotenoid metabolic networks. Our findings confirmed regional environment was also the determinant of metabolic patterns. Distinct production regions (WT, FY, and CZ) exhibited 33, 31, and 32 carotenoid metabolites, respectively, demonstrating unique region-specific distribution patterns. Differential metabolite analysis revealed significant upregulation trend in WT, with zeaxanthin palmitate and violaxanthin emerging as regionally dominant high-abundance carotenoids. Consequently, geographic biomarkers such as zeaxanthin-myristate-palmitate can be interpreted as end products of G×E-specific metabolic reprogramming. Taken together, the region-specific metabolic signatures identified here are not static but represent plastic outcomes of genotype-by-environment interactions, wherein environmental effects are consistently modulated by genetic background.

Although a formal pathway enrichment analysis was beyond the scope of this ecological study, the differential metabolites identified offer direct insight into underlying biosynthetic processes. The recurrent detection of zeaxanthin-myristate-palmitate as a cross-regional biomarker underscores the xanthophyll esterification pathway as environmentally responsive. The accumulation of specific esters such as zeaxanthin palmitate and violaxanthin myristate in regions like WT suggests environmental influences on acyltransferase activity or substrate availability, potentially enhancing carotenoid stability [59, 60]. Concurrent variations in violaxanthin and its esters further indicate modulation of the violaxanthin cycle and downstream branching pathways. These region-specific metabolite profiles align with the known plasticity of carotenoid biosynthesis under abiotic factors [48, 58], thereby pinpointing key pathway nodes most sensitive to G×E interactions. These metabolites are important ingredients of functional food, demonstrating efficacy in retinal protection and anti-inflammatory immunomodulation [16]. This metabolic signature appears correlates strongly with WT’s unique ecological conditions, characterized by higher altitude (~ 1200 m), lower mean annual temperature, and relatively abundant rainfall. These environmental factors may modulate transcriptional activity of key biosynthetic enzymes, particularly LCYB (lycopene β-cyclase) and BCH (β-carotene hydroxylase), thereby promoting the accumulation of specific metabolites [10, 48]. These results reinforced that geographical origin is a primary determinant of crop metabolite profiles, where greater environmental disparities correspond to more pronounced metabolic differences [66–68].

Our results demonstrate that acylated xanthophylls (notably zeaxanthin-myristate-palmitate) as production-region biomarkers, enabling novel approaches for geographical provenance verification. Although the present study did not include gene expression analysis, the observed geographic and genotypic variations in carotenoid profiles suggest potential differential regulation of key biosynthetic genes such as *PSY* and *CCD*. Future studies combining transcriptomics and genetic engineering will be essential to validate these hypotheses and uncover the molecular basis of G×E interactions in foxtail millet, enabling precision breeding of region-specific nutraceutical crops.

‌Systematic analysis of carotenoid metabolome variations across different regions not only facilitated the identification of region-specific functional metabolites, but also provided practical guidance for establishing premium production zones and developing high-value functional food development. It should be noted that the generalizability of these findings may be influenced by the study’s focus on limit cultivars within a specific regional context. To build upon this work, future studies incorporating a wider genetic diversity, expanded geographical range, and multi-year observations will be essential to validate and generalize these models. Furthermore, integrating multi-omics with geospatial analytics could elucidate the molecular mechanisms underlying genotype-by-environment interactions, thereby accelerating the development of climate-resilient cultivars.

## Conclusion

This study demonstrates that optimal foxtail millet quality, characterized by high kernel yellowness and carotenoid content, was achieved in regions with moderate effective accumulated temperatures, balanced precipitation, and fertile soils rich in phosphorus and organic matter. GGE biplot analysis revealed distinct genotype-by-environment interactions. It showed that high-performing but environmentally sensitive cultivars are best suited to favorable regions, while more stable cultivars perform better in marginal environments. Through metabolomic profiling, zeaxanthin-myristate-palmitate was identified as a geographic biomarker. Based on these findings, we recommend implementing precision cultivar zoning guided by GGE patterns. We also suggest adopting agronomic practices such as adjusting sowing dates and applying phosphorus or organic fertilizers in suboptimal regions to improve foxtail millet quality. This research provides a theoretical foundation for foxtail millet breeding and regional cultivation strategies. Building on this work, the systematic integration of genomics with metabolomics and the development of environment-specific prediction models will enable the precise selection of climate-resilient cultivars.

## Supplementary Information

Supplementary Material 1.

## Data Availability

Data will be made available on request.

## Abbreviations

BVK: b value of kernel
BVP: b value of powder
HADT: Daily maximum temperature
LADT: Daily minimum temperature, ADT: daily average temperature
PRE: Precipitation
ARH: Air relative humidity
DTR: Diurnal temperature range
SSD: Sunshine duration
Alt: Altitude
AN: Available nitrogen
AP: Available phosphorus
AK: Available potassium
SOM: Soil organic matter
TN: Total nitrogen
TP: Total phosphorus
Ca: Available calcium
Mg: Available magnesium
Fe: Available iron
B: Available boron. GGE: genotype plus genotype-by-environment interaction
OPLS-DA: Orthogonal partial least squares-discriminant analysis
RDA: Redundancy analysis
HR: Huairen
WT: Wutai
YP: Yuanping
DX: Dingxiang
GJ: Gujiao
YC: Yuci
FY: Fenyang
QX: Qinxian
CZ: Changzhi
YQ: Yuanqu
UPLC-MS/MS: Ultra-Performance Liquid Chromatography-Tandem Mass Spectrometry method
