# Supplementary material for: Genotype × environment interactions underlying geographic divergence in carotenoid accumulation and kernel pigmentation of foxtail millet
Source: BMC Plant Biol. 2026 Jan 2;26:201. doi: 10.1186/s12870-025-08008-1 (PMC12866036; doi:10.1186/s12870-025-08008-1)
Supplement: Supplementary file 1 — Supplementary Material 1. [file 12870_2025_8008_MOESM1_ESM.docx]

**Table S1** Information of foxtail millet samples

| Code | Cultivar | Origin | Accession Number |
| --- | --- | --- | --- |
| G1 | Jingu21 | Institute of Economic Crops, Shanxi Academy of Agricultural Sciences | SXAU-FM-JG21 |
| G2 | Changlong47 | Institute of Millet Crops, Shanxi Academy of Agricultural Sciences | SXAU-FM-CL47 |
| G3 | Qinhuang2 | Shanxi Qinzhouhuang Millet (Group) Co., Ltd. | SXAU-FM-QH2 |
| G4 | Jinfen107 | Institute of Economic Crops, Shanxi Academy of Agricultural Sciences | SXAU-FM-JF107 |
| G5 | Yugu18 | Anyang Academy of Agricultural Sciences, Henan Province | SXAU-FM-YG18 |
| G6 | Jixiang1 | Institute of Millet Crops, Hebei Academy of Agriculture and Forestry Sciences | SXAU-FM-JX1 |
| G7 | Zhangza10 | Zhangjiakou Academy of Agricultural Sciences, Hebei Province | SXAU-FM-ZZ10 |
| G8 | Jigu21 | Crop Research Institute, Shandong Academy of Agricultural Sciences | SXAU-FM-JIG21 |
| G9 | Zhonggu2 | Institute of Crop Sciences, Chinese Academy of Agricultural Sciences | SXAU-FM-ZG2 |
| G10 | Zhonggu19 | Institute of Crop Sciences, Chinese Academy of Agricultural Sciences | SXAU-FM-ZG19 |
| G11 | Baigu9 | Baicheng Academy of Agricultural Sciences, Jilin Province | SXAU-FM-BG9 |
| G12 | Jinmiao K1 | Millet Research Institute, Chifeng Academy of Agricultural Sciences | SXAU-FM-JMK1 |

**Table S2** Geographic information of sampling sites

| Code | Location | Latitude /N° | Longitude/E° | Altitude/m |
| --- | --- | --- | --- | --- |
| HR | Huairen county, Shuozhou city, Shanxi province | 39.93 | 113.28 | 1039 |
| WT | Wutai county, Xinzhou city, Shanxi province | 38.8 | 113.68 | 1154 |
| YP | Yuanping county, Xinzhou city, Shanxi province | 38.77 | 112.72 | 815 |
| DX | Dingxiang county, Xinzhou city, Shanxi province | 38.59 | 112.88 | 768.4 |
| GJ | Gujiao county, Taiyuan city, Shanxi province | 37.98 | 112.25 | 1324 |
| YC | Yuci county, Jinzhong city, Shanxi province | 37.55 | 112.67 | 799 |
| FY | Fenyang county, Lvliang city, Shanxi province | 37.25 | 111.78 | 767.7 |
| QX | Qinxian county, Changzhi city, Shanxi province | 36.73 | 112.66 | 1002.5 |
| CZ | Luzhou county, Changzhi city, Shanxi province | 36.20 | 113.13 | 932.70 |
| YQ | Yuanqu county, Yuncheng city, Shanxi province | 35.20 | 111.62 | 710.00 |

**Table S3**Combined ANOVA for color parameters and carotenoid content

| Indicator | Source | SS | df | MS | F-value | P-value | Contribution rate(%) |
| --- | --- | --- | --- | --- | --- | --- | --- |
| BVK | G | 366.31 | 11 | 33.30 | 16.65 | 0.001 | 58.65 |
|  | E | 106.85 | 9 | 11.87 | 4.28 | 0.001 | 17.11 |
|  | G×E | 151.42 | 99 | 1.53 | 1.70 | 0.044 | 24.24 |
|  | Error | 183.21 | 240 |  |  |  |  |
|  | Total | 897.79 | 359 |  |  |  |  |
| BVP | G | 536.67 | 11 | 48.79 | 37.31 | 0.001 | 67.71 |
|  | E | 125.04 | 9 | 13.89 | 10.65 | 0.001 | 15.78 |
|  | G×E | 130.85 | 99 | 1.32 | 1.52 | 0.144 | 16.51 |
|  | Error | 180.14 | 240 |  |  |  |  |
|  | Total | 254.43 | 359 |  |  |  |  |
| Carotenoid | G | 163.55 | 11 | 14.87 | 19.39 | 0.001 | 53.39 |
|  | E | 61.90 | 9 | 6.88 | 4.33 | 0.001 | 20.21 |
|  | G×E | 80.88 | 99 | 0.82 | 1.33 | 0.037 | 26.40 |
|  | Error | 39.55 | 240 |  |  |  |  |
|  | Total | 86.471 | 359 |  |  |  |  |

G: genotype (cultivar); E: environment (location); SS: sum of squares; df: degrees of freedom; MS: mean square; Contribution rate = SS_F_/ (SS_G_ + SS_E_ +SS_G×E_); df_G_ = N_G_ -1 =11; df_E_ =N_E_ - 1 = 9; df_G×E_ = (N_G_ -1) × (N_E_ - 1) = 99; df_total_ = N_G_ × N_E_ × 3 (replicates) - 1 = 359; df_error_ = df_total_ - df_G_ - df_E_ - df_G×E_ = 240.

**Table S4** Soil chemical properties across ecological sites

| Year | Code | Location | pH | AN | AP | AK | SOM | TN | TP | Ca | Mg | Fe | B |
| --- | --- | --- | --- | --- | --- | --- | --- | --- | --- | --- | --- | --- | --- |
| 2022 | E1 | HR | 8.47 | 41.1 | 22.60 | 176.36 | 10.91 | 0.715 | 0.868 | 1.204 | 134.4 | 2.90 | 0.266 |
|  | E2 | WT | 7.44 | 88.2 | 39.47 | 140.00 | 14.4 | 1.159 | 1.041 | 1.121 | 86.8 | 9.65 | 0.133 |
|  | E3 | YP | 8.22 | 58.4 | 31.21 | 195.64 | 17.3 | 0.748 | 0.988 | 1.657 | 161.0 | 2.88 | 0.143 |
|  | E4 | DX | 8.46 | 41.1 | 18.60 | 216.75 | 9.08 | 0.700 | 0.855 | 1.288 | 88.6 | 1.92 | 0.241 |
|  | E5 | GJ | 8.66 | 39.0 | 20.04 | 149.69 | 9.41 | 0.730 | 0.833 | 1.406 | 114.4 | 1.32 | 0.084 |
|  | E6 | YC | 8.60 | 49.3 | 31.04 | 193.22 | 25.9 | 1.355 | 1.259 | 1.635 | 220.9 | 0.80 | 0.440 |
|  | E7 | FY | 8.51 | 51.9 | 35.15 | 232.20 | 17.3 | 1.048 | 1.025 | 1.678 | 260.7 | 1.05 | 0.599 |
|  | E8 | QX | 8.45 | 47.5 | 22.91 | 199.79 | 14.2 | 0.826 | 0.802 | 1.565 | 49.1 | 1.84 | 0.112 |
|  | E9 | CZ | 8.23 | 71.3 | 22.94 | 219.99 | 28.8 | 1.511 | 1.046 | 1.944 | 143.6 | 3.00 | 0.221 |
|  | E10 | YQ | 7.79 | 80.0 | 20.39 | 223.92 | 25.0 | 1.400 | 1.025 | 1.448 | 145.8 | 6.16 | 0.089 |
| 2023 | E11 | HR | 8.22 | 48.14 | 36.90 | 184.71 | 13.12 | 0.850 | 1.124 | 0.897 | 123.4 | 2.14 | 0.59 |
|  | E12 | WT | 7.35 | 61.72 | 37.14 | 135.23 | 14.79 | 0.900 | 1.110 | 0.992 | 82.4 | 9.73 | 0.58 |
|  | E13 | YP | 8.08 | 53.69 | 33.36 | 134.97 | 17.52 | 0.838 | 1.246 | 1.583 | 135.4 | 2.62 | 0.10 |
|  | E14 | DX | 8.29 | 46.90 | 20.16 | 218.37 | 12.14 | 0.801 | 0.995 | 0.995 | 80.0 | 2.05 | 0.49 |
|  | E15 | GJ | 8.71 | 34.56 | 22.73 | 234.12 | 9.48 | 0.579 | 0.869 | 1.343 | 184.4 | 0.88 | 0.12 |
|  | E16 | YC | 8.69 | 61.72 | 42.94 | 246.78 | 21.32 | 1.289 | 1.381 | 1.211 | 203.6 | 0.60 | 0.48 |
|  | E17 | FY | 8.31 | 62.33 | 39.29 | 189.15 | 20.33 | 1.227 | 1.273 | 1.515 | 213.9 | 1.43 | 0.35 |
|  | E18 | QX | 8.39 | 85.17 | 41.07 | 237.36 | 23.21 | 1.492 | 1.121 | 2.199 | 112.9 | 1.86 | 0.09 |
|  | E19 | CZ | 7.95 | 83.93 | 22.02 | 170.84 | 28.23 | 1.622 | 1.345 | 2.141 | 113.0 | 5.09 | 0.10 |
|  | E20 | YQ | 7.47 | 60.48 | 15.21 | 129.40 | 13.61 | 0.953 | 0.811 | 1.948 | 119.0 | 6.32 | 0.54 |

AN: available nitrogen (mg/kg); AP: available phosphorus (mg/kg); AK: available potassium (mg/kg); SOM: soil organic matter (g/kg), TN: total nitrogen (g/kg); TP: total phosphorus (g/kg); Ca: available calcium (g/kg), Mg: available magnesium (mg/kg), Fe: available iron (mg/kg), B: available boron (mg/kg).

**Table S5**Meteorological characteristics across ecological sites

| Year | Code | Location | HADT | LADT | ADT | DTR | EAT | PRE | ADR | SSD |
| --- | --- | --- | --- | --- | --- | --- | --- | --- | --- | --- |
| 2022 | E1 | HR | 24.96 | 12.69 | 18.74 | 12.28 | 1338.60 | 403.03 | 53.30 | 896.77 |
|  | E2 | WT | 23.37 | 13.72 | 18.44 | 9.65 | 1304.46 | 731.15 | 69.97 | 834.52 |
|  | E3 | YP | 25.30 | 13.65 | 19.56 | 11.66 | 1470.13 | 555.95 | 62.47 | 846.14 |
|  | E4 | DX | 23.34 | 13.18 | 18.19 | 10.16 | 1272.79 | 557.37 | 65.30 | 845.93 |
|  | E5 | GJ | 24.14 | 13.23 | 18.53 | 10.91 | 1322.67 | 677.27 | 62.35 | 849.44 |
|  | E6 | YC | 27.09 | 16.09 | 21.27 | 11.00 | 1728.09 | 425.35 | 58.39 | 849.56 |
|  | E7 | FY | 26.59 | 15.61 | 20.94 | 10.98 | 1677.08 | 463.48 | 60.05 | 848.01 |
|  | E8 | QX | 26.06 | 15.33 | 20.47 | 10.73 | 1606.98 | 324.24 | 63.54 | 829.65 |
|  | E9 | CZ | 25.54 | 15.36 | 20.17 | 10.18 | 1566.37 | 377.79 | 65.02 | 841.60 |
|  | E10 | YQ | 26.59 | 17.70 | 21.99 | 8.89 | 1836.85 | 456.50 | 67.46 | 829.85 |
|  | AVG |  | 25.30 | 14.66 | 19.83 | 10.64 | 1512.40 | 497.21 | 62.79 | 847.15 |
| 2023 | E11 | HR | 25.32 | 13.29 | 19.33 | 12.03 | 1429.05 | 321.32 | 52.80 | 901.64 |
|  | E12 | WT | 23.56 | 14.00 | 18.83 | 9.56 | 1352.25 | 676.56 | 70.55 | 836.62 |
|  | E13 | YP | 25.49 | 14.22 | 19.96 | 11.27 | 1525.72 | 410.22 | 62.52 | 858.81 |
|  | E14 | DX | 24.78 | 14.21 | 19.43 | 10.57 | 1445.87 | 457.08 | 66.35 | 842.79 |
|  | E15 | GJ | 24.22 | 13.61 | 18.81 | 10.61 | 1352.84 | 532.38 | 63.13 | 851.71 |
|  | E16 | YC | 27.52 | 16.40 | 21.74 | 11.12 | 1797.83 | 370.93 | 58.43 | 852.32 |
|  | E17 | FY | 26.83 | 15.90 | 21.18 | 10.94 | 1712.47 | 345.56 | 60.16 | 835.26 |
|  | E18 | QX | 26.84 | 15.95 | 21.22 | 10.89 | 1718.08 | 295.67 | 61.75 | 811.81 |
|  | E19 | CZ | 26.03 | 15.98 | 20.69 | 10.05 | 1638.39 | 368.50 | 65.78 | 825.83 |
|  | E20 | YQ | 25.99 | 17.72 | 21.75 | 8.27 | 1797.36 | 539.16 | 73.92 | 763.77 |
|  | AVG |  | 25.66 | 15.13 | 20.29 | 10.53 | 1576.99 | 431.74 | 63.54 | 838.06 |

HADT: daily maximum temperature (℃); LADT: daily minimum temperature (℃); ADT: daily average temperature (℃); DTR: diurnal temperature range (℃); EAT: effective accumulated temperature (℃); PRE: precipitation (mm); ARH: air relative humidity (%); SSD: sunshine duration (h).

**Table S6** Differential carotenoid metabolites among sample groups

| Index | B1vsA1 | C1vsA1 | C1vsB1 | B2vsA2 | C2vsA2 | C2vsB2 |
| --- | --- | --- | --- | --- | --- | --- |
| α-carotene |  | down | down | up |  | down |
| β-carotene |  |  |  |  |  |  |
| ε-carotene |  |  |  |  |  |  |
| antheraxanthin dipalmitate |  | up | up | up |  | down |
| lutein caprate |  |  |  | down | down |  |
| lutein laurate |  | down | down | up |  | down |
| lutein palmitate |  |  |  |  |  |  |
| lutein stearate |  |  |  |  |  |  |
| 5,6epoxy-lutein-caprate-palmitate | up | up |  |  |  |  |
| lutein dipalmitate |  |  |  |  |  |  |
| lutein oleate |  |  |  |  |  |  |
| neochrome palmitate |  |  |  |  |  |  |
| violaxanthin dibutyrate |  |  | up | down | down | down |
| violaxanthin laurate | down | down |  |  |  |  |
| violaxanthin myristate |  |  |  |  |  |  |
| violaxanthin palmitate |  |  |  |  |  |  |
| violaxanthin palmitoleate |  |  |  |  |  |  |
| violaxanthin dilaurate | up | up |  |  |  |  |
| violaxanthin-myristate-caprate |  |  |  |  |  |  |
| violaxanthin-myristate-palmitate |  |  |  |  |  |  |
| violaxanthin dipalmitate |  | up | up | up |  |  |
| violaxanthin dioleate |  |  |  | down | down |  |
| zeaxanthin palmitate |  |  |  |  |  |  |
| zeaxanthin dilaurate |  | up | up |  | up |  |
| zeaxanthin dimyristate |  |  |  |  |  |  |
| zeaxanthin-laurate-palmitate | down | down |  |  |  |  |
| zeaxanthin-myristate-palmitate | down | down | up | up | up | down |
| zeaxanthin dipalmitate |  |  |  |  |  |  |
| zeaxanthin-palmitate-stearate |  |  |  |  | down | down |
| zeaxanthin-oleate-palmitate |  | down | down |  | up |  |
| antheraxanthin |  |  |  |  |  |  |
| zeaxanthin |  |  |  |  |  |  |
| violaxanthin |  |  | up | down |  |  |
| neoxanthin |  | up | up |  |  |  |
| lutein |  |  |  |  |  |  |
| β-cryptoxanthin |  |  |  | down |  |  |
| α-cryptoxanthin | down |  |  | down |  |  |
| echinenone |  |  |  | down | down |  |
| β-citraurin |  |  |  |  | up |  |

up: up-regulated metabolites; down: down-regulated metabolites.


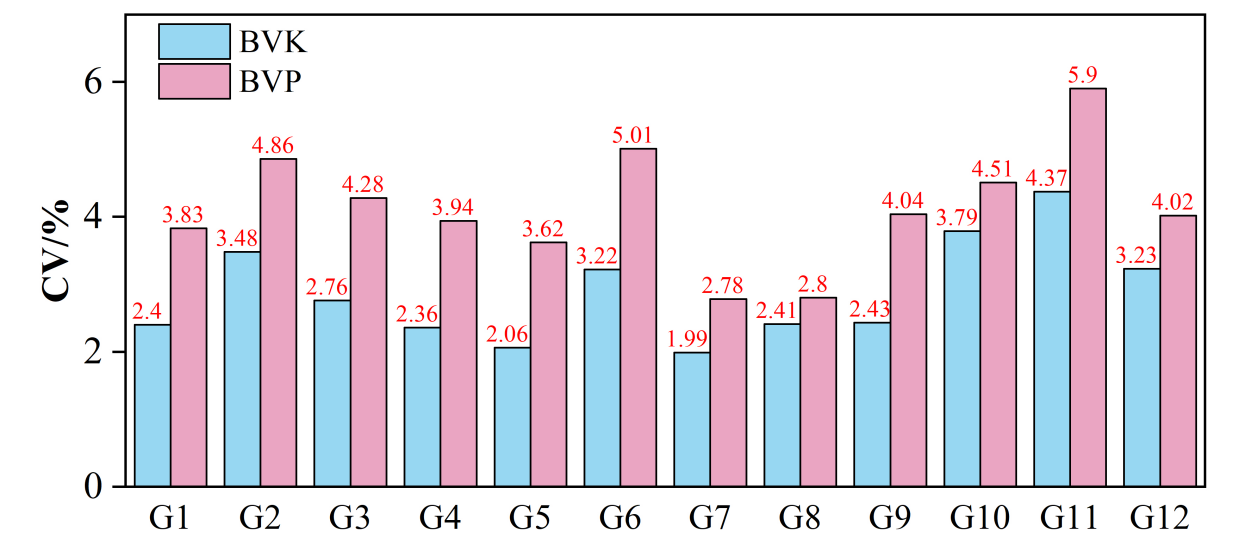

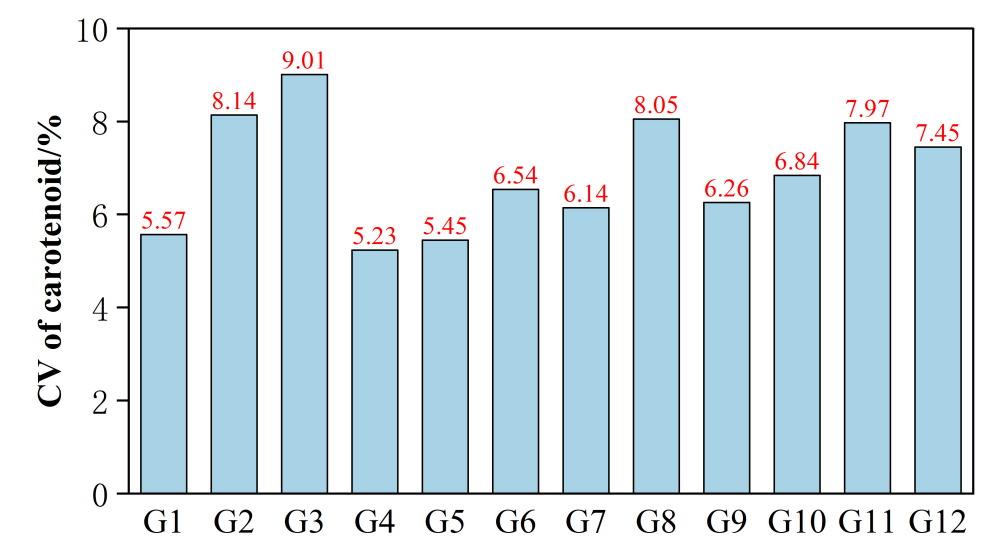


**A**

**B**

**Fig. S1.** A: Variation degree of BVK and BVP among different cultivars. B: Variation degree of carotenoid contents among different cultivars. BVK: b value of kernel; BVP: b value of powder; CV: coefficients of variation.


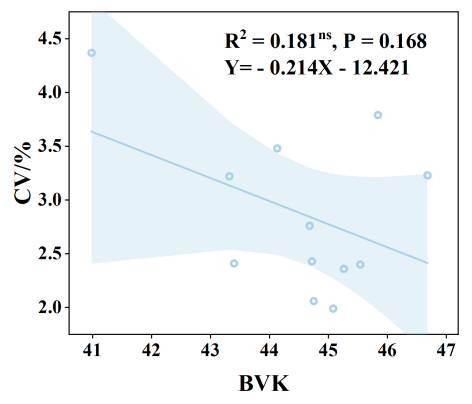

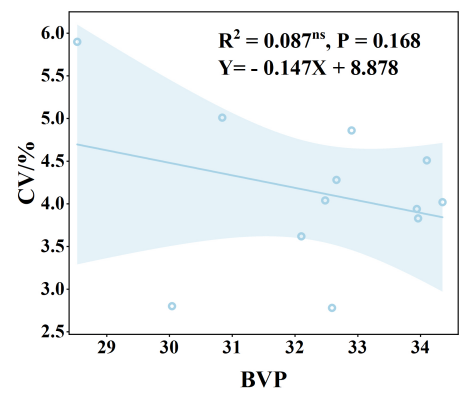

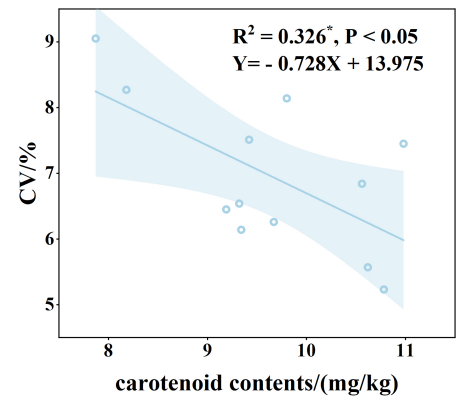


**A**

**B**

**C**

**Fig. S2.** Correlation analysis of the coefficient of variation with BVK (A), BVP (B), and carotenoid contents (C). BVK: b value of kernel; BVP: b value of powder; CV: coefficients of variation.
